# Evaluating the effect of denoising submillimeter auditory fMRI data with NORDIC

**DOI:** 10.1162/imag_a_00270

**Published:** 2024-08-14

**Authors:** Lonike K. Faes, Agustin Lage-Castellanos, Giancarlo Valente, Zidan Yu, Martijn A. Cloos, Luca Vizioli, Steen Moeller, Essa Yacoub, Federico De Martino

**Affiliations:** Department of Cognitive Neuroscience, Faculty of Psychology and Neuroscience, Maastricht University, Maastricht, The Netherlands; Department of Neuroinformatics, Cuban Neuroscience Center, Havana City, Cuba; Center for Advanced Imaging Innovation and Research (CAI2R), Department of Radiology, New York University School of Medicine, New York, NY, United States; Bernard and Irene Schwartz Center for Biomedical Imaging, Department of Radiology, New York University School of Medicine, New York, NY, United States; MRI Research Center, University of Hawaii, Honolulu, HI, United States; Australian Institute for Bioengineering and Nanotechnology, University of Queensland, St. Lucia, Australia; Center for Magnetic Resonance Research, Department of Radiology, University of Minnesota, Minneapolis, MN, United States

**Keywords:** fMRI, auditory neuroscience, denoising, high-resolution, NORDIC

## Abstract

Functional magnetic resonance imaging (fMRI) has emerged as an essential tool for exploring human brain function. Submillimeter fMRI, in particular, has emerged as a tool to study mesoscopic computations. The inherently low signal-to-noise ratio (SNR) at submillimeter resolutions warrants the use of denoising approaches tailored at reducing thermal noise—the dominant contributing noise component in high-resolution fMRI. NOise Reduction with DIstribution Corrected Principal Component Analysis (NORDIC PCA) is one of such approaches, and has been benchmarked against other approaches in several applications. Here, we investigate the effects that two versions of NORDIC denoising have on auditory submillimeter data. While investigating auditory functional responses poses unique challenges, we anticipated NORDIC to have a positive impact on the data on the basis of previous applications. Our results show that NORDIC denoising improves the detection sensitivity and the reliability of estimates in submillimeter auditory fMRI data. These effects can be explained by the reduction of the noise-induced signal variability. However, we did observe a reduction in the average response amplitude (percent signal change) within regions of interest, which may suggest that a portion of the signal of interest, which could not be distinguished from general i.i.d. noise, was also removed. We conclude that, while evaluating the effects of the signal reduction induced by NORDIC may be necessary for each application, using NORDIC in high-resolution auditory fMRI studies may be advantageous because of the large reduction in variability of the estimated responses.

## 1 Introduction

In recent years, the use of ultra-high field (UHF) magnetic resonance imaging (MRI) has rapidly increased for a variety of applications. At UHF, the signal-to-noise ratio (SNR -Vaughan et al., 2001) and the blood-oxygenation-level-dependent (BOLD) contrast (Ogawa et al., 1992), the basis of functional MRI (fMRI), increase (Yacoub et al., 2001). This results in higher sensitivity to fMRI responses compared to more conventional field strengths (e.g., 3T and below). This allows for enormous benefits for the study of human brain function, in particular, the ability to acquire high spatial resolution (below 1 mm isotropic voxels) images. As such, at UHF it is possible to breach into the mesoscale and investigate fundamental computational structures and organizations of cortical functions, such as layers and columns (see e.g.De Martino et al., 2018;Dumoulin et al., 2018;Huber et al., 2015;Kok et al., 2016;Lawrence et al., 2019;Moerel et al., 2021;Olman et al., 2012;Uğurbil, 2018;Yacoub et al., 2008;Zimmermann et al., 2011).

The functional contrast to noise ratio (fCNR) in fMRI is dependent on the signal change compared to baseline and both physiological and thermal noise. Submillimeter fMRI at UHF trades the higher SNR for spatial resolution, often leaving the resulting data in a thermal noise dominated regime (characterized as unstructured, zero-mean Gaussian distributed noise) emanating from electrical sources inherent to MRI hardware (Triantafyllou et al., 2005,^2011^). This makes approaches oriented towards reducing thermal noise (i.e., improving the image SNR) of particular interest for neuroscience applications that require mesoscopic level imaging. Importantly, approaches for improving image SNR have to be evaluated against any practical considerations or tradeoffs, for example, their ability to preserve spatial information content at the finest scales (e.g., laminar and columnar cortical responses -Polimeni et al., 2018) or whether any unwanted biases are introduced (Kay, 2022). Extensive averaging, one of the possible approaches for reducing thermal noise, could, in principle, help in highlighting small functional changes without altering the signal content. However, this approach—which assumes constant responses to the same stimuli over extended periods of time—is limited by practical considerations such as the overall length of scanning sessions and the need for aligning data across multiple imaging sessions. While precision imaging approaches, that collect extensive data in only a few individuals, are becoming increasingly interesting in particular settings (see, e.g.,Allen et al., 2022;Michon et al., 2022;Poldrack et al., 2017), their application to mesoscopic imaging is far from standard and may not suffice when questions are oriented to generalizing effects at the population level. As an alternative to averaging, spatial smoothing could be used to increase image SNR. However, its application needs careful consideration as it comes with inevitable loss of spatial specificity (Turner & Geyer, 2014), which can be controlled if it is combined with anatomically informed constraints (e.g., laminar smoothing maintains specificity in the cortical depth direction while smoothing only tangentially -Huber et al., 2021;Kiebel et al., 2000). Apart from averaging and (image) smoothing (with anatomical constraints), approaches for improving the detectability of effects (i.e., overcoming the limitations of low SNR regimes) have been considered at the analysis stage. Multivariate analyses, for example, have been argued to better leverage the information present in fine-grained patterns and, in part, overcome the lower SNR of high-resolution functional images, but may have some limitations in interpretability (Formisano & Kriegeskorte, 2012). In univariate analyses, the definition of noise regressors (through, e.g., principal component analysis -Kay et al., 2013) has also been considered in order to improve the detectability of effects of interest, but relies on knowledge of the experimental design and assumptions such as the definition of noise pools (i.e., a collection of voxels whose time series is mostly representing noise sources). Denoising based on independent component analysis (ICA) has also been developed in fMRI and evaluated primarily in its ability to remove structured noise components (Pruim et al., 2015) and improving detectability of effects in lower-resolution functional data that are mainly challenged by physiological noise (Griffanti et al., 2014;Salimi-Khorshidi et al., 2014). For completeness, it is important to note that approaches to remove structured (physiological) noise in fMRI (and thus not tailored to the reduction of thermal noise) include, apart from ICA, the use of multiple echoes to estimate sources of variance (Gonzalez-Castillo et al., 2016;Steel et al., 2022), including nuisance regressors in the general linear model (e.g., CompCor -Behzadi et al., 2007), or measuring physiological data to subsequently remove the noise sources from the data (e.g., RETROICOR -Glover et al., 2000; or RETROKCOR -Hu et al., 1995).

A denoising technique tailored to the removal of thermal noise that has recently been introduced is NOise Reduction with DIstribution Corrected Principal Component Analysis (NORDIC PCA -Moeller et al., 2021;Vizioli et al., 2021). NORDIC is a pre-processing approach based on PCA that selectively removes components that are indistinguishable from zero-mean normally distributed noise (see, e.g.,https://layerfmri.com/2023/07/10/nordic/#more-3956for an informal description of the approach). Compared to other PCA denoising techniques (seeVeraart et al., 2016), the main difference rests in the approach used to estimate the number of (principal) components that are removed (i.e., the threshold on the eigenvalue spectrum that distinguishes noise components from signal components). NORDIC has been initially extensively evaluated on visual cortical responses elicited by blocked (temporally prolonged ~ 12 seconds) stimulation and has been shown to increase detection sensitivity without affecting the overall signal change and spatial precision of the responses (i.e., without introducing spatial blurring -Vizioli et al., 2021). NORDIC has also been evaluated and compared to other PCA-based denoising approaches (dwidenoise -Cordero-Grande et al., 2019;Manzano Patron et al., 2024;Veraart et al., 2016). Compared to dwidenoise and more conventional smoothing approaches, and in experimental designs ranging from blocked to event-related visual stimulation, NORDIC has been shown to better preserve local and global spatial smoothness of the functional data as well as the temporal characteristics of the responses (i.e., temporal smoothing) and it has been shown to not introduce unwanted effects (Dowdle et al., 2023; but seeFernandes et al., 2023for an evaluation in rodent data). NORDIC has been rapidly picked up by the community and its usability is now being examined across different areas (including visual and motor regions), field strengths (3T and 7T), and acquisition techniques (see, e.g.,Dowdle et al., 2022;Knudsen et al., 2023;Raimondo et al., 2023). These recent studies consistently show that NORDIC improves detectability of the effects in single-subject data. However, while NORDIC has been shown to improve (statistical) signal detection, generalizing these results to other cortical regions and to designs that are particularly SNR limited (e.g., auditory cortical responses elicited by slow event-related designs) still requires careful evaluation of its benefits as opposed to any potential unwanted bias.

Here, we focus on the application of NORDIC to submillimeter fMRI data collected to investigate auditory cortical responses elicited by a slow event-related design. The auditory cortex is located next to large air cavities with parts of it, like primary cortical regions, lying further away from the receive coils compared to other sensory regions (e.g., visual and somatosensory regions). These and other factors (e.g., the need for large field of views to image bilateral auditory cortical areas) make imaging auditory cortical regions sensitive to geometric distortions and signal dropouts due to large B_0_inhomogeneities (Moerel et al., 2021) and not only for BOLD-type acquisitions (Faes et al., 2023). Furthermore, the percent signal change elicited in auditory regions is lower than in the visual cortex (De Martino et al., 2015). However, despite these challenges, there have been several high-resolution auditory studies that look at cortical depth-dependent responses (see, e.g.,Ahveninen et al., 2016;De Martino et al., 2015;Gau et al., 2020;Moerel et al., 2018). As such, given that auditory submillimeter studies are especially restricted by low SNR, they would greatly benefit from thermal noise reduction. However, the efficacy of PCA-based denoising methods also depends on the relative contribution of signal and noise. Therefore, submillimeter auditory fMRI may present a challenge for NORDIC. Collectively, these considerations warrant the need to explore the effect of NORDIC denoising in the auditory cortex—going beyond evaluations in visual and somatosensory areas. Following previous NORDIC evaluations, we consider the effects of NORDIC on tSNR and follow-up by focusing on changes of beta estimates, t-statistics and their spatial reliability before and after NORDIC. As our evaluation is based on submillimeter data, we also offer a preliminary investigation of laminar profiles in selected regions of the temporal lobe.

## 2 Methods

### 2.1 NORDIC

NORDIC is a denoising approach that operates on either complex-valued or magnitude-only fMRI time series. As the use of parallel imaging results in a spatially varying amplification of the thermal noise characterized by the g-factor, if necessary, the NORDIC algorithm first normalizes the functional data by the estimated g-factor (Ma et al., 2020), resulting in the thermal noise being uniformly distributed across space to fulfill the assumption of PCA denoising that noise is identically distributed across voxels. The g-factor reduces SNR due to parallel imaging and that depends on the geometry of the coil (for further clarification seePruessmann et al. 1999). NORDIC uses a locally low rank approach to perform a patch-wise PCA across space and time. An estimate of the absolute noise level is obtained from an appended acquisition without a radiofrequency excitation (e.g., a noise scan) to account for the difference between the estimated and true g-factor. In each patch, the noise threshold defines the principal components that are removed from the eigenspectrum as they are considered to be indistinguishable from zero-mean Gaussian distributed noise. The noise threshold is chosen with Monte-Carlo simulations for a Casorati matrix with zero-mean normally distributed sampling and, depending on the settings, considering a theoretical or estimated noise distribution. After the removal of noisy principal components, the patches are recombined and the g-factor is re-applied to reconstruct the fMRI images. Assuming signal redundancy within the patch (i.e., enough voxels carrying the same information, i.e., the 4D time series is locally low-rank), NORDIC aims to remove thermal noise from the time series while preserving the fine-grained temporal and spatial structure of the signal that is assumed to be carried by the preserved principal components. For more details on NORDIC we refer to the original publications (Moeller et al., 2021;Vizioli et al., 2021).

Currently, there are two implementations of NORDIC available. Here, we focus on the use of NIFTI_NORDIC, which takes nifti formatted data of both magnitude and phase images as input (for the specific version we have used, seehttps://github.com/SteenMoeller/NORDIC_Raw/, commit c96acf9).

### 2.2 MR imaging acquisition

Data were collected with a 7T Siemens Magnetom System with a single-channel transmit and 32-channel receive NOVA head coil (Siemens Medical Systems, Erlangen). Whole-brain anatomical T1-weighted images were collected using a Magnetisation Prepared 2 Rapid Acquisition Gradient Echo (MP2RAGE) sequence at a resolution of 0.75 mm isotropic (192 slices, TR = 4300 ms, TE = 2.27 ms) (Marques et al., 2010).

Functional data were acquired with 2D gradient-echo (GE) echo planar imaging (EPI) along with simultaneous multi-slice/multiband (Moeller et al., 2010;Setsompop et al., 2012) (0.8 mm isotropic, 42 slices, TR = 1600 ms, TE = 26.4 ms, MB factor 2, iPAT factor 3, 6/8 Partial Fourier, bandwidth 1190 Hz, field of view: 170 × 170 mm, matrix size: 212 × 212, phase encoding = anterior to posterior; coil combination = SENSE1).

### 2.3 Participants

Ten healthy participants took part in this fMRI study (aged between 23 and 69 years old, 5 females). Participants had no history of neurological disease or hearing disorders. Eight participants were scanned at the Center for Magnetic Resonance Research in Minneapolis (CMRR) and two were scanned at New York University (NYU) using the identical imaging protocol except for slight differences in TR (TR_CMRR_= 1600 ms, TR_NYU_= 1650 ms). The local IRB at the individual institutions approved the experiment. All participants signed informed consent forms before commencing the study.

### 2.4 Experimental design

Participants passively listened to tone sequences. Conditions were designed to investigate predictive processing in the auditory cortex (based on sequences used inBerlot et al., 2018), but we will disregard the neuroscientific purpose of the experimental paradigm and focus on the effect of denoising instead.

Six conditions were presented. All conditions consisted of sequences of four tones. The four tones were presented for 100 ms each with a 400 ms gap between tones (total tone sequence length was 1.6 seconds). The conditions were designed such that the first three tones were ‘contextual’ tones ordered in either a descending, ascending or scrambled fashion. The frequencies used for these contextual tones were always the same three (493.9, 659.3 and 987.8 Hz), albeit presented in a different order. The fourth tone was selected such that three conditions ended in a high frequency (1318.5 Hz) and three conditions ended in a low frequency (329.6 Hz). This resulted in two predictable sequences (PredH and PredL), two mispredicted sequences (MispredH and MispredL), and two unpredictable sequences (UnpredH and UnpredL) as displayed inFigure 1. The auditory stimuli were presented concomitantly with the scanner noise (i.e., no silent gap for sound presentation was used).

**Fig. 1.**
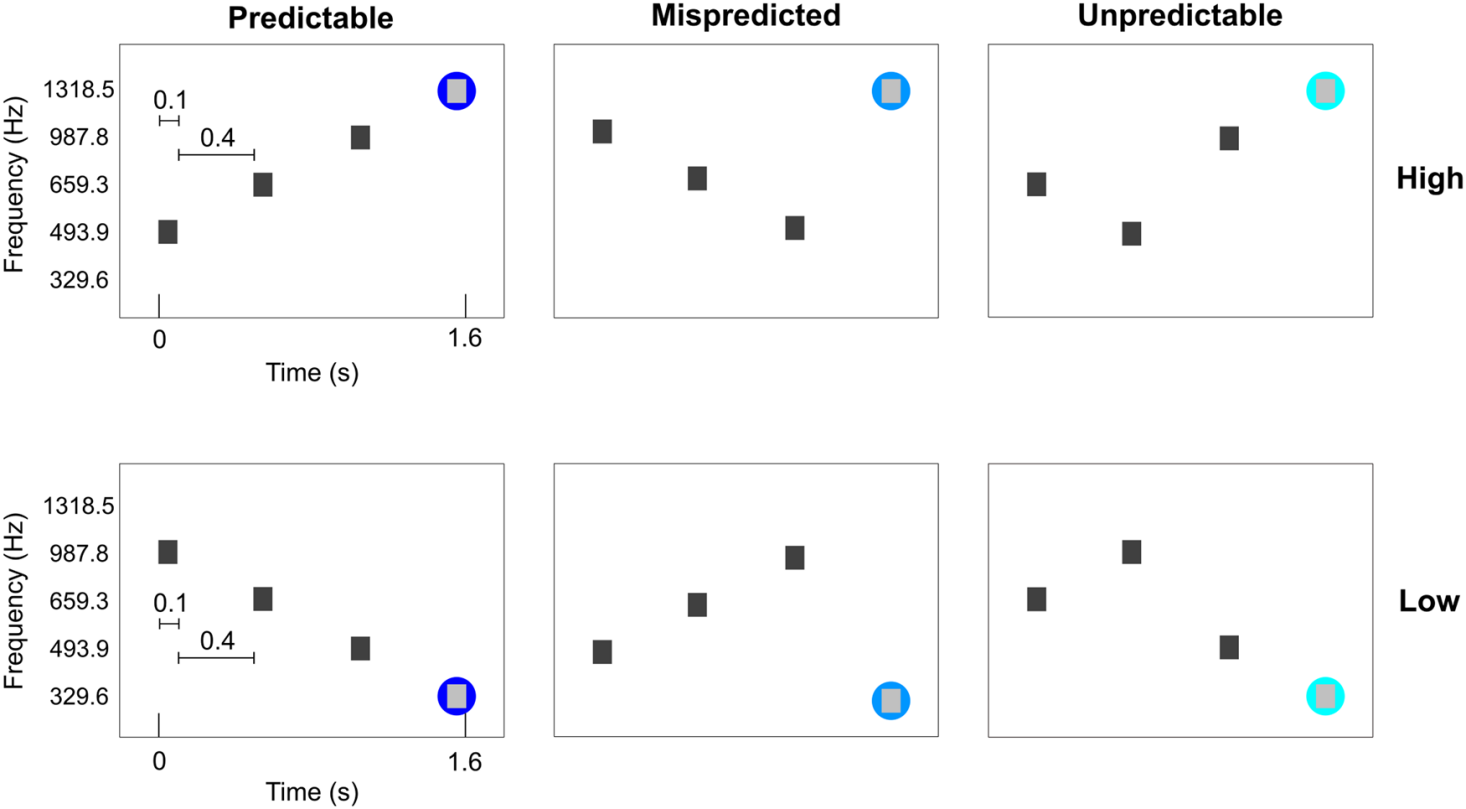
Experimental conditions. The first three tones are contextual eliciting a strong or weak prediction. The three contextual tones of each condition are presented at the same frequencies, albeit in different orders. The fourth tone could be a high or low target frequency. The fourth tone can either consecutively follow the ascending or descending order (PredH and PredL), it could be deviant (MispredH and MispredL) or the contextual tones could be scrambled before presenting the target frequency (UnpredH and UnpredL). The two predictable sequences were presented ten times per run, whereas the other four conditions were presented four times per run. Please note that the precise timing of these conditions is illustrated for the data collected at CMRR. Data collected at NYU used a 50 ms longer TR, while stimuli were always presented time locked to the TR number.

Per run, each of the predictable sequences were presented 10 times; the mispredicted sequences and the unpredictable sequences were each presented 4 times in a randomized order (for a total of 36 trials in one run). Tone sequences were presented in a slow event-related design with an average inter-trial interval of 6 TR’s (ranging between 5 and 7 TR’s). For each participant, we collected 6 to 8 runs that lasted approximately 6 minutes each (consisting of 230 volumes of functional data and five additional noise scans at the end of each run at CMRR and 229 volumes of functional data and one additional noise scan at NYU). Magnitude and phase Dicom images were exported from the scanner. It is important to note that the experimental task did not differ across collection sites.Figure 1illustrates the timing of stimulus presentation with a TR of 1.6 seconds (TR_CMRR_). Stimuli were presented always locked to the TR number (not absolute time or relative time with respect to the start of the experiment). The only difference in data collection between sites was that, at NYU, the TR was 50 ms longer (TR_NYU_= 1.65 seconds).

### 2.5 Data preprocessing

#### 2.5.1 NORDIC preprocessing

Dicom files were converted to NIfTI format, separately for magnitude and phase images (MRIcron, version 1.0.20190902). The magnitude and phase NifTI files were used as the input to NIFTI_NORDIC (seeFig. S1for magnitude and phase images before and after NORDIC). We used two different settings for NORDIC denoising: 1) using default settings for fMRI data (a ratio in spatial to temporal dimensions for the PCA kernel size of 11:1, removal of a low-resolution volume specific phase estimate (temporal phase = 1), low-resolution phase filter with a Tukey-filter (scale-factor 10), and the noise scan was used for empirical noise estimation) and 2) the same as the default except for the use of the noise scan in the estimation of the noise threshold (seehttps://github.com/SteenMoeller/NORDIC_Raw/for further explanation of the meaning of each setting). In the NIFTI_NORDIC implementation, not using the noise scan results in using a noise threshold based on the g-factor estimation (in the implementation, a threshold of 1/sqrt(2)), which is generally more conservative (i.e., resulting in the removal of less principal components) than the estimated threshold when using the noise scan, reflecting the empirical observation that the approach for g-factor estimation underestimates the value by up to 10%. This resulted in three datasets (per run), the first, which will be referred to as the*‘Original’*, represents the fMRI data without NORDIC denoising. The second, which we will refer to as*‘NORDIC default’*(NORdef), represents the fMRI time series resulting from the processing with default NORDIC settings. The third, we will refer to as ‘*NORDIC No Noise*’ (NORnn), represents the fMRI time series resulting from the use of NORDIC without separate noise scans for the estimation of the noise threshold.

#### 2.5.2 Pre-processing

The anatomical and functional data were analyzed using a BrainVoyager software package (BV – version 21.4 unless otherwise specified, Brain Innovation, Maastricht, The Netherlands) and custom Matlab scripts (The MATHWORKS Inc., Natick, MA, USA). After the initial NORDIC denoising step, functional processing was performed identically across datasets. First, the noise acquisitions were removed from each time series. Pre-processing of the functional data included slice scan time correction using sinc interpolation and motion correction along three dimensions using intrasession alignment to the run closest in time to the collection of opposite phase encoding images (run 1 in most participants, run 4 in two participants). In addition, temporal filtering was applied to remove low frequencies (high-pass filtering with 7 cycles per run) and high frequencies (temporal gaussian smoothing with a full width half maximum kernel of 2 data points). Reversed phase polarity acquisitions were used to correct for geometric distortions using Topup (FSL version 6.0.4). In one participant, we experienced issues collecting opposed phase polarity images and therefore no distortion correction was performed in this participant.

The anatomical data were upsampled in image space to 0.4 mm isotropic, corrected for inhomogeneities and transformed to ACPC space. A segmentation was created using the deep neural network in BV (version 22.0) to determine the initial white matter (WM) and gray matter (GM) boundary and GM/cerebral spinal fluid (CSF) border. The segmentation of the temporal lobe was manually corrected in ITK snap (version 3.8.0 -Yushkevich et al., 2006). With this corrected segmentation, we created mid-GM surface meshes in BV. Additionally, we estimated the cortical thickness of the high-resolution segmentation.

#### 2.5.3 ROI definition

Five bilateral regions of interest (ROIs) were manually drawn on the individual mid-GM meshes based on macro-anatomical landmarks (as described inKim et al., 2000), covering the temporal lobe including Heschl’s Gyrus (HG), Planum Polare (PP), Planum Temporale (PT), anterior superior temporal gyrus (aSTG), and posterior superior temporal gyrus (pSTG). The delineation of ROIs on one hemisphere of an exemplary participant can be found inFigure S2. These ROIs were projected back onto the anatomy in volume space (extending 3 mm inwards and outwards from the mid-GM surface). These masks were first intersected with the GM definition to create GM-confined ROIs and then dilated (six steps) in order to obtain the final masks that include GM as well as the WM and the CSF surrounding it. The union of all the masks (temporal lobe mask) was used to run the statistical analysis (General Linear Model, see below), while results were inspected separately per ROI in some analyses.

### 2.6 Analyses

#### 2.6.1 General linear model

All statistical analyses were performed with custom Matlab scripts. Time series were first normalized to percent signal change (PSC). For our first-level analysis, we fitted a general linear model (GLM) with single trials per condition as predictors (36 trials and one constant per run). Predictors were convolved with a standard two-gamma hemodynamic response function (HRF) that peaked at 5 seconds after the onset of the stimuli. In order to evaluate the effects that NORDIC has on the reliability of the responses, we obtained response estimates (beta weights) and computed statistical activation maps by considering the variability across single trials (i.e., beta time series) for all predictors combined (sounds versus no sounds) and for each condition separately (please note that PSC and betas are interchangeable in the text as the design matrix was scaled to one and the HRF kernel sums to one). In other words, we here estimate the variance of the response considering the variability across trials and not the variance of the residuals of the GLM fit. This helped us in evaluating measures of reliability of the response estimates.

After the GLM, in each individual’s anatomical ROI (considering all voxels in the ROI) we evaluated: 1) the change in beta per condition before and after NORDIC processing; 2) the change in single-trial t-statistics (mean divided by variance across trials); 3) the spatial replicability of the mean betas; and 4) the spatial replicability of the t-statistics. For all individual subject data, all analyses were performed by randomly sampling half of the runs (i.e., repeated split half with 50 repetitions -Valente et al., 2021). The spatial replicability of the betas and t-statistics was computed by performing a voxel-wise correlation of the variable of interest (beta or t-statistics) across the two random splits of the data in each ROI separately. Finally, across all ROIs we investigated changes in beta values (before and after NORDIC) in relation to the tSNR. Note that we compute tSNR (defined as the mean divided by the standard deviation of the time series) on the Original data after pre-processing (tSNR_pr_). This choice inflates the tSNR we report compared to the more conventional choice to calculate tSNR on the un-preprocessed data (in analyses not shown, we confirmed that the results we report here are not dependent on the choice or pre-processing applied to the time series).

At the group level, interactions were tested with repeated-measures ANOVA (where processing strategy is the repeated measure). Main effects were tested for significance using permutation testing by permuting, for each test, individual subject data across processing strategies (all possible permutations [2^10^]) and corrected for multiple comparisons using Bonferroni.

#### 2.6.2 Correlation and cross-validation analyses

To evaluate the spatial similarity of beta estimates across processing strategies, we computed the correlation of the estimated beta maps in the broad temporal lobe mask. In particular, we considered: 1) the correlation of each NORDIC processed run (NORdef and NORnn) to the corresponding original run (separately for each of the six conditions); 2) the run-to-run correlation within each processing strategy (i.e., within Original, NORdef and NORnn data); and 3) using leave-one-run-out (LORO), the correlation of each run (i.e., run 3) to the average of all other runs (all runs except run 3). Importantly, for this last analysis, the reference model (i.e,. the averaged map coming from all runs except one) to which the left-out run of Original NORdef and NORnn data was compared was extracted from the original time series (i.e., we did not compute an average map of the NORdef or NORnn time series).

At the group level, interactions (e.g., processing strategy and condition in the first analysis) were tested with repeated-measures ANOVA (where processing strategy is the repeated measure). To do this, data were Fisher z-transformed prior to the ANOVA. Main effects were tested for significance using permutation testing by permuting, for each test, individual subject data across processing strategies (all possible permutations [2^10^]) and corrected for multiple comparisons using Bonferroni.

#### 2.6.3 Tonotopic maps

From the two predictable conditions we created tonotopic maps (as best frequency maps, seeFormisano et al., 2003;Heynckes et al., 2023for an example where the procedure is applied with only two frequencies, as is the case here). Tonotopic maps were computed in volume space and interpolated to the mid-cortical surface.

#### 2.6.4 Variance partitioning

We reasoned that the total variance from the original (magnitude) time series (per voxel) could be partitioned as follows:

$$Y_{ori}\;=\text{​}\alpha {\text{Y}}_{AN}\;\;+b+\varepsilon_{AN}$$

Where${𝑌}_{ori}$and${𝑌}_{𝐴𝑁}$are the original time series and the time series after NORDIC preprocessing respectively (${𝑌}_{𝐴𝑁}$can then come from either NORdef or NORnn). By estimating the scaling factor and intercept ($\hat{\alpha }$and$\hat{b}$) with ordinary least squares (OLS), we obtained estimated residuals (${\hat{\varepsilon }}_{AN}$) that represent the portion of the original time series that is orthogonal to the NORDIC time series (i.e., our approach is equivalent to Gram-Shmidt orthogonalization). We refer to${\hat{\varepsilon }}_{AN}$as the residuals of the original time series after NORDIC (residuals after NORDIC in short). This decomposition guarantees that the total sum of squares of the Original data (representing the variability in the data with respect to their mean) can be expressed as the sum of squares of the data after NORDIC (weighted by$\hat{\alpha }$) and the sum of squares portion of the Original data that is orthogonal to the data processed with NORDIC (i.e., the residuals after NORDIC${\hat{\varepsilon }}_{AN}$). That is:

$$SSY_{ori}={\hat{\alpha }}^{2}SSY_{AN}+SSY_{{\hat{\varepsilon }}_{AN}}$$

To quantify the variance associated with the experimental design in the Original data, as well as the data after NORDIC processing and the residuals after NORDIC (${\hat{\varepsilon }}_{AN}$), we regressed${𝑌}_{ori}$,${𝑌}_{𝐴𝑁}$, and${\hat{\varepsilon }}_{AN}$against our design matrix (X). This second regression allowed us to partition the variance that, in each of the three signals of interest (Y*_ori_*,${𝑌}_{𝐴𝑁}$, and${\hat{\varepsilon }}_{AN}$), is related to the design ($SSY_{\;\;\;\;ori}^{X}$,$SSY_{\;\;\;\;AN}^{X}$,$SSY_{\;\;\;\;{\hat{\varepsilon }}_{AN}}^{X}$), along with an error term for each.

We present the results by calculating the ratio of the sum of squares. First, within each processing strategy (Original, NORdef, and NORnn), we compared the variance explained by the design to the total variance of each respective time series. Second, for the NORDIC processed data (NORdef and NORnn) we compared the variance associated with the design, in their respective residuals after NORDIC (${\hat{\varepsilon }}_{AN\_NORdef},$${\hat{\varepsilon }}_{AN\_NORnn}$), to the total sum of squares of the original time series. This last analysis allowed us to reveal the portion of the variance associated with the design that is not present in the NORDIC processed data and is thus removed by NORDIC.

#### 2.6.5 Laminar analysis

We explored the effect of NORDIC denoising on the cortical depth dependent estimates. Beta maps were computed across 11 cortical depths and sampled on the mid-GM surface in BV. These maps were subsequently intersected with a mask of HG. The single-trial betas were averaged across vertices within HG in each depth and subsequently across trials. The variability was computed across trials.

## 3 Results

### 3.1 Activation and spatial patterns

**Fig. 2.**
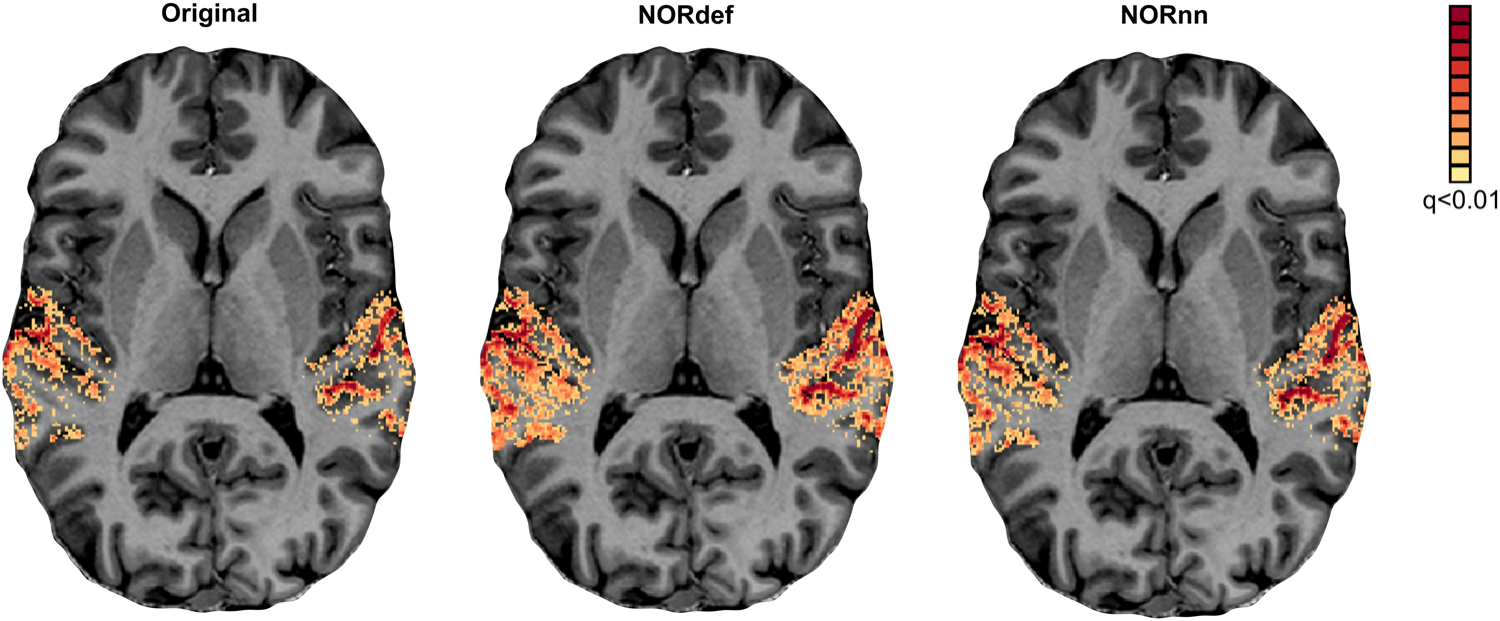
Single-subject overall response to sounds (qFDR<0.01). From left to right, we show the t-maps resulting from a GLM with single trials as predictors of the Original, NORdef, and NORnn data on a transversal slice. NORdef: default setting, including a noise scan to estimate noise threshold, NORnn: same settings except for the use of noise scan.

We assessed the effect of NORDIC on detection sensitivity by evaluating the overall activation (sounds > no sounds) elicited by single trials in our experimental design. Statistical maps were computed by considering the mean and variability (t-statistic) across single trials (not the GLM residuals) and corrected for multiple comparisons using a false discovery rate of qFDR<0.01. This is a more stringent threshold than the customary qFDR<0.05 because it allows better visual appreciation of the differences between processing strategies in each individual.Figure 2presents the results in one exemplary volunteer on a representative transversal anatomical slice, highlighting the statistical advantage in detection sensitivity in single-subject data conferred by denoising (as shown before in, e.g.,Dowdle et al., 2023;Vizioli et al., 2021). At the same statistical threshold, both NORdef and NORnn resulted in more significant voxels. In this volunteer, for example, 34% of voxels in our temporal lobe mask were significantly active at the qFDR threshold, whereas NORdef and NORnn resulted in 51% and 44% of voxels active, respectively. NORDIC denoising resulted in overall higher t-statistics; the 90th percentile across voxels for each of three datasets was 10.38, 13.42, and 12.38, respectively. These results are in line with previous applications of NORDIC (Dowdle et al., 2022,^2023^;Knudsen et al., 2023;Raimondo et al., 2023;Vizioli et al., 2021) and similar to these previous reports, activation maps do not appear spatially distorted (i.e., blurred) when comparing NORDIC processed data to the original.

**Fig. 3.**
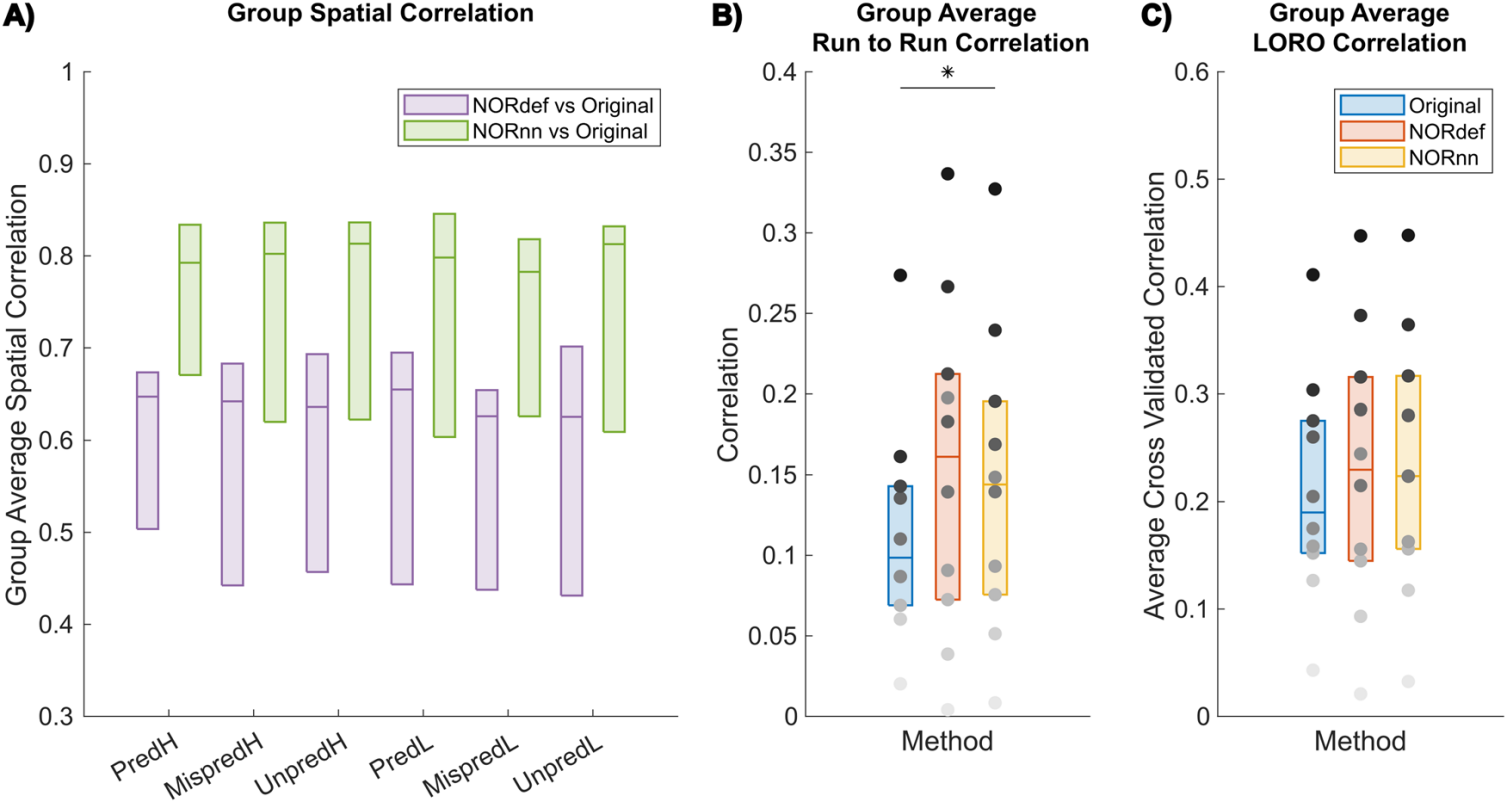
Group average correlation analyses. (A) NORnn is more similar to the Original data than NORdef. However, across conditions, there is no evidence of a difference between correlation values between the NORDIC denoised datasets and the Original data (two-way repeated-measures ANOVA showed no main effect of condition). This indicates that the number of repetitions of each condition do not affect the effect of NORDIC denoising. (B) The run-to-run stability of beta estimates increases significantly with the use of NORnn, that is, across subjects, beta estimates are more similar across runs after NORnn. (C) Average cross-validated correlation values of single runs to the average of the Original data for the PredH condition. Grayscale dots indicate mean correlation values of individual participants ordered according to their correlation value in the Original data (participants have the same color in each of the three datasets). * indicates p < 0.05.

To evaluate some of these effects further, we analyzed the spatial patterns of activation separately per condition.Figure 3Ashows the group average of the similarity (correlation) of beta maps averaged across trials per condition of NORdef and NORnn to the beta maps obtained from the Original data considering the variability across the mean estimates of every subject (all single-subject figures are shown inFig. S3A). Correlation coefficients were compared with a two-way repeated measures ANOVA (with condition and processing strategy as factors). There was no interaction between condition and processing strategy (p = 0.536). Permutation testing showed a main effect of method (p < 0.001), indicating that at the group level NORnn results in a larger similarity of the spatial patterns to the Original data. This is in line with NORnn being more conservative, that is, resembling more the Original data (due to a lower noise threshold and the removal of less noise components). However, the correlation values were similar across conditions (no significant main effect, p = 0.122). That is, the similarity was not influenced by the different amount of repetitions of specific conditions (e.g., the mispredicted and unpredictable conditions). Therefore, in what follows, we present results of the predictable condition(s) only.

Figure 3Bshows the average run-to-run reproducibility across participants of the spatial patterns of activation within each processing strategy for PredH (for individual subject figures seeFigs. S3B and S4Bfor an alternative visualization of single-subject data points). The stability of run-to-run estimates (Fig. 3B) was significantly higher for the NORnn compared to the Original data (p = 0.041), whereas there was no evidence of a difference between NORdef and the Original data (p = 0.064). Interestingly, subjects that showed low run-to-run correlation in the Original data did not improve after NORDIC denoising (grayscale dots indicate mean values of individual participants).

Lastly, in the absence of a ground truth, we reasoned that the spatial pattern elicited by averaging multiple runs of the Original data would be a reasonable choice to compare the results of single runs in their ability to approximate results obtained with higher SNR. To this end, we computed the average of the spatial pattern of activation elicited by the PredH condition in the Original data in all but one run. This reference pattern was correlated to the left-out run in the Original data and to the same run after NORDIC processing (leave-one-run-out - LORO). We repeated this analysis each time leaving a different run out. At the group level, the correlation of a single run to the average of our reference was not significant in either NORdef compared to the Original data (p = 0.258) or NORnn compared to the Original data (p = 0.053). To appreciate the individual differences, all single-subject figures are available inFigure S3Cand seeFigure S4Cfor an alternative visualization of single-subject data points.

**Fig. 4.**
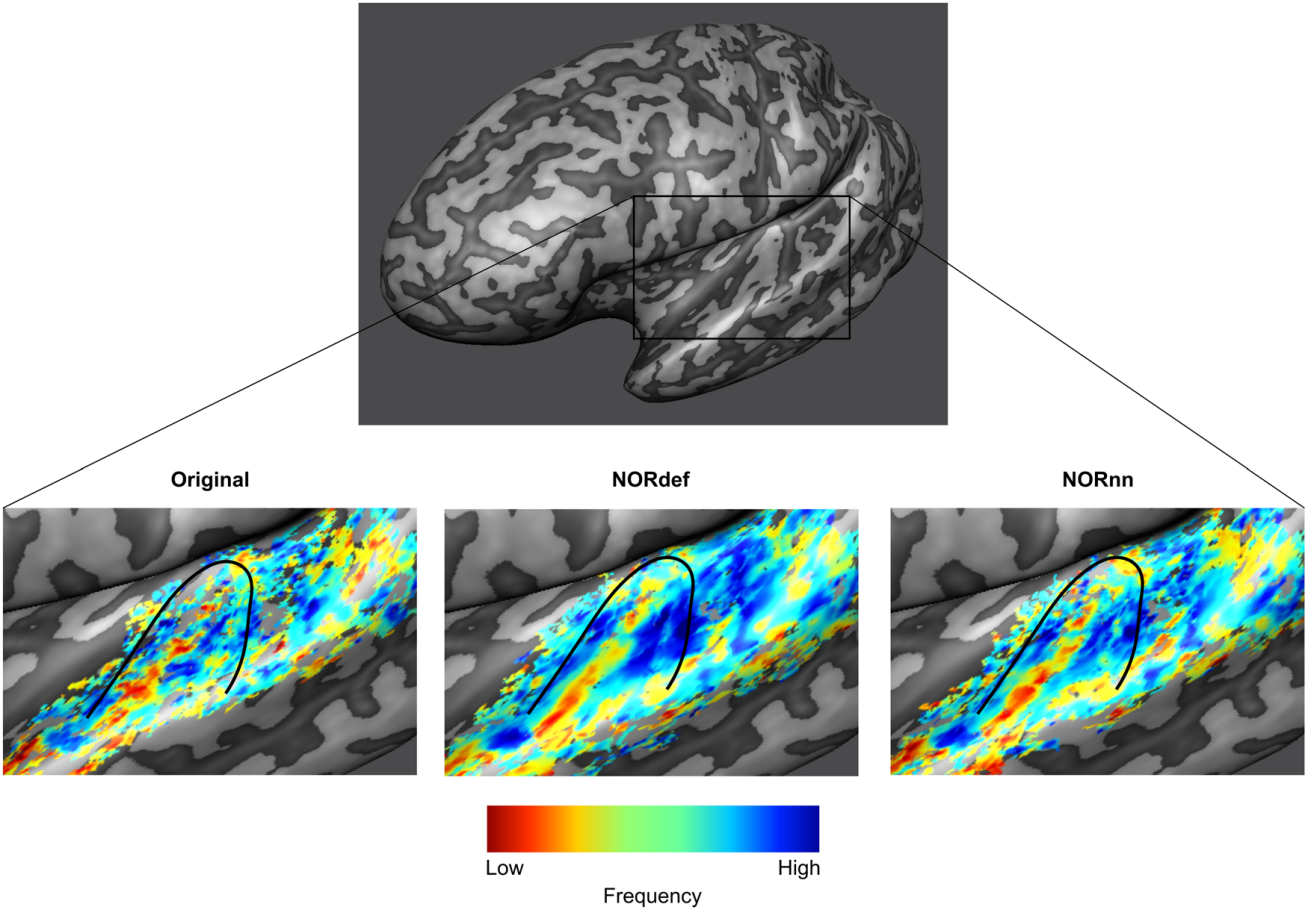
Example tonotopic maps. Frequency preference maps are computed for each dataset and for one example participant we display these maps on an inflated mid-GM surface. Denoising does not seem to alter the frequency preference as the high-low-high gradient is visible in all three datasets. The maps computed from the denoised datasets are less noisy. The black line delineates HG.

Our design also allows the derivation of tonotopic maps, albeit from only two frequencies, by computing best frequency maps on the PredH and PredL conditions.Figure 4shows, for an exemplary single left hemisphere, tonotopic maps projected on the mid-GM surface intersected with their respective t-map. As expected (Moerel et al., 2014), a low-frequency preferring region is visible along Heschl’s gyrus (HG) surrounded by two high-frequency preferring areas. This gradient is visible in the Original data and becomes more discernible in the NORnn and NORdef tonotopic maps, respectively. The fact that some regions are more clearly preferring one of the two frequencies (i.e., blue regions anterior to HG) highlights the fact that after NORDIC the frequency preference is more spatially homogeneous and less corrupted by noise. This figure serves as an illustration of what to expect when NORDIC denoising is used in single-subject studies looking at tonotopic maps.

**Fig. 5.**
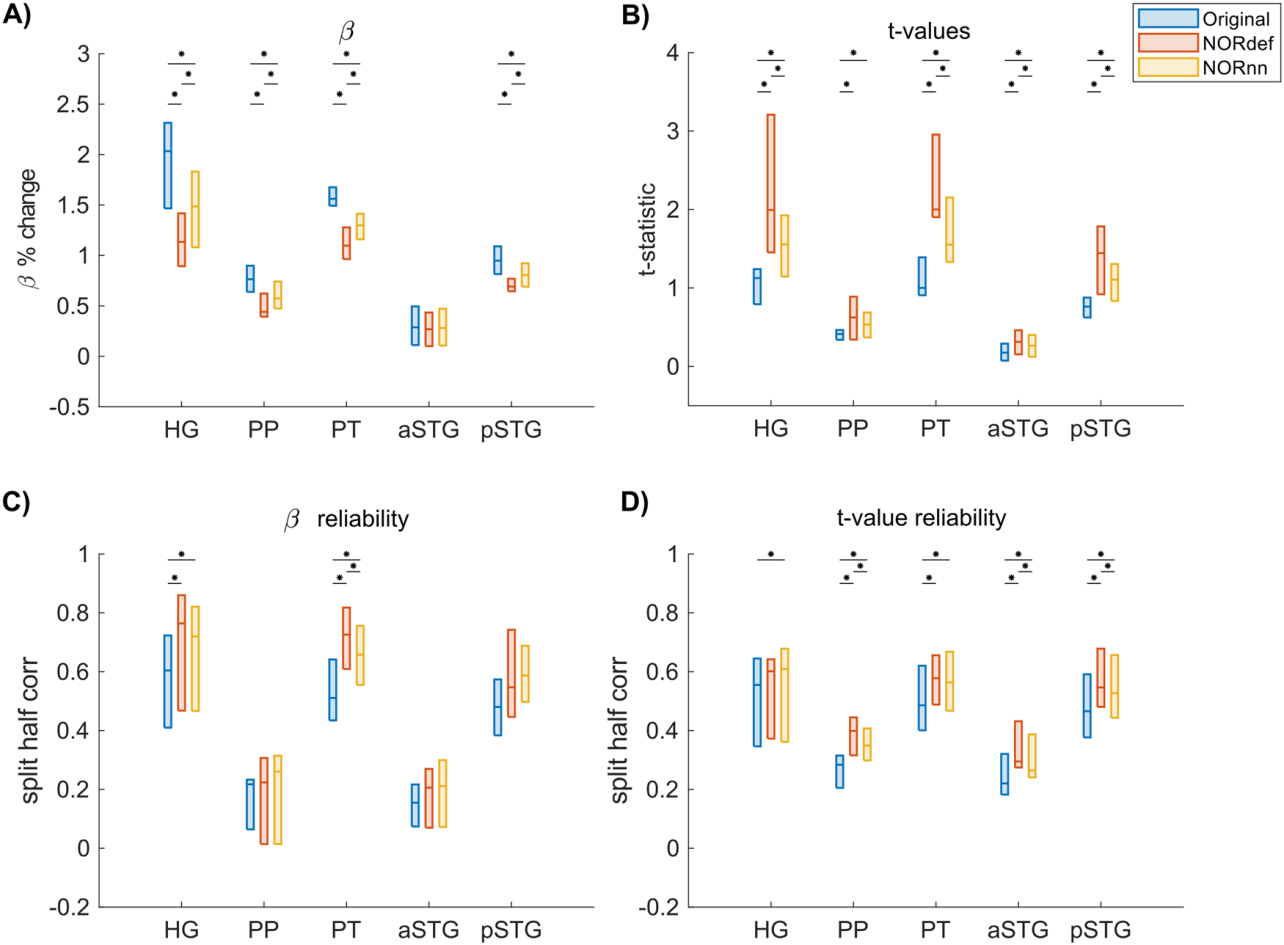
Beta- and t-value estimates and their reliability. (A) Average reduction of beta values across participants. (B) At the group level, in increase in t-values is visible. (C) On average, denoising results in a better estimate of beta values calculated with split half correlations in ROIs where there is more signal in the data. (D) t-value reliability is generally higher after NORDIC than in the Original data. * indicates p < 0.05.

The previous applications of NORDIC (Dowdle et al., 2023;Vizioli et al., 2021) have shown that detection sensitivity with NORDIC comes due to a reduction in variance without any change to the percent signal response. While this effect would explain our results at the level of the whole temporal lobe (reported inFig. 3), we also investigated changes in percent signal as well as its variability across trials in separate anatomically defined ROIs. In the temporal lobe, in most ROIs, NORDIC denoising resulted in reduced betas (Figs. 5AandS5for individual subjects,Figure S9Afor the group plot with lines indicating single-subject data). This reduction was more pronounced in the NORdef compared to NORnn. Within each subject, reductions in betas though come with a larger reduction in variability of the response across trials when using NORDIC. This is clear when considering t-values within each of the ROIs (Figs. 5BandS6for individual subjects,Fig. S9Bfor the group plot with lines indicating single subject data). The increase in t-values is most apparent in the NORdef time series. These changes induced by NORDIC processing are visible in ROIs that are activated by our design (i.e., the pattern is less visible in the aSTG that has little activation in our experiment). The change in betas induced by NORDIC is most evident in voxels whose overall signal level is low (seeFigs. 6andS10for individual subjects). However, the biases introduced by NORDIC in the single ROIs do not come with detrimental effects to the reliability of the estimates in each ROI compared to the analysis at the level of the whole temporal lobe. When analyzing the reliability of spatial patterns in the individual ROIs (Figs. 5CandS7, and5DandS8for betas and t-values, respectively. See alsoFigs. S9C and S9Dfor the group plot with lines indicating single subject data), NORDIC denoising is associated with a general improvement in reliability. These results indicate that within each subject, in our data, there is evidence for a bias-variance tradeoff associated with the application of NORDIC. Repeated-measures ANOVAs showed a significant interaction between processing strategy and ROI for each of the subfigures ofFigure 5(all Greenhouse-Geisser corrected p-values were smaller than 0.05). The resulting p-values of the permutation tests (Bonferroni corrected) can be found inTable 1of theSupplementary Materials.

**Fig. 6.**
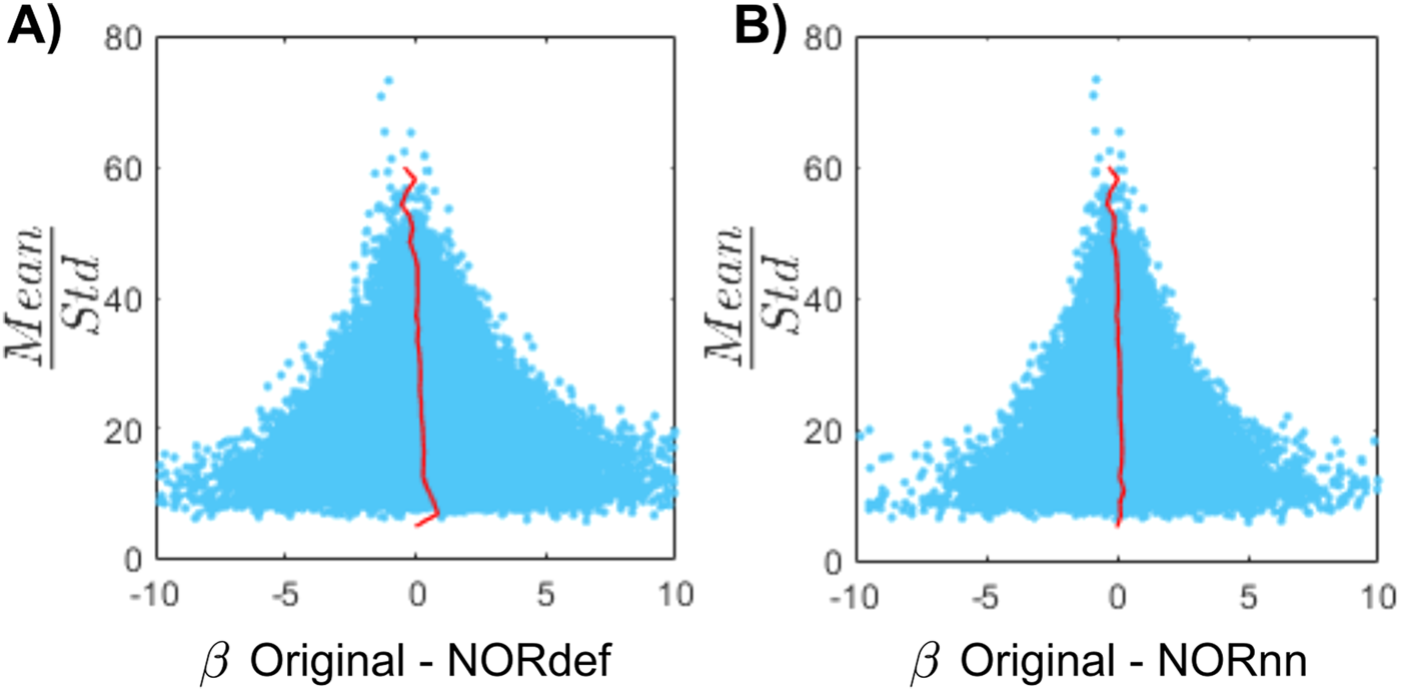
Beta difference in relation to tSNR for one representative subject. (A) Mean/standard deviation (tSNR_pr_) is displayed as a function of the Original data and betas after NORDIC. For low tSNR_pr_values, the betas change in both directions. However, at high tSNR_pr_, the betas remain relatively similar after NORDIC. The red line indicates the mean beta difference per bin. (B) Same as (A) but for the beta difference between Original and NORnn betas.

### 3.2 Variance explained

**Fig. 7.**
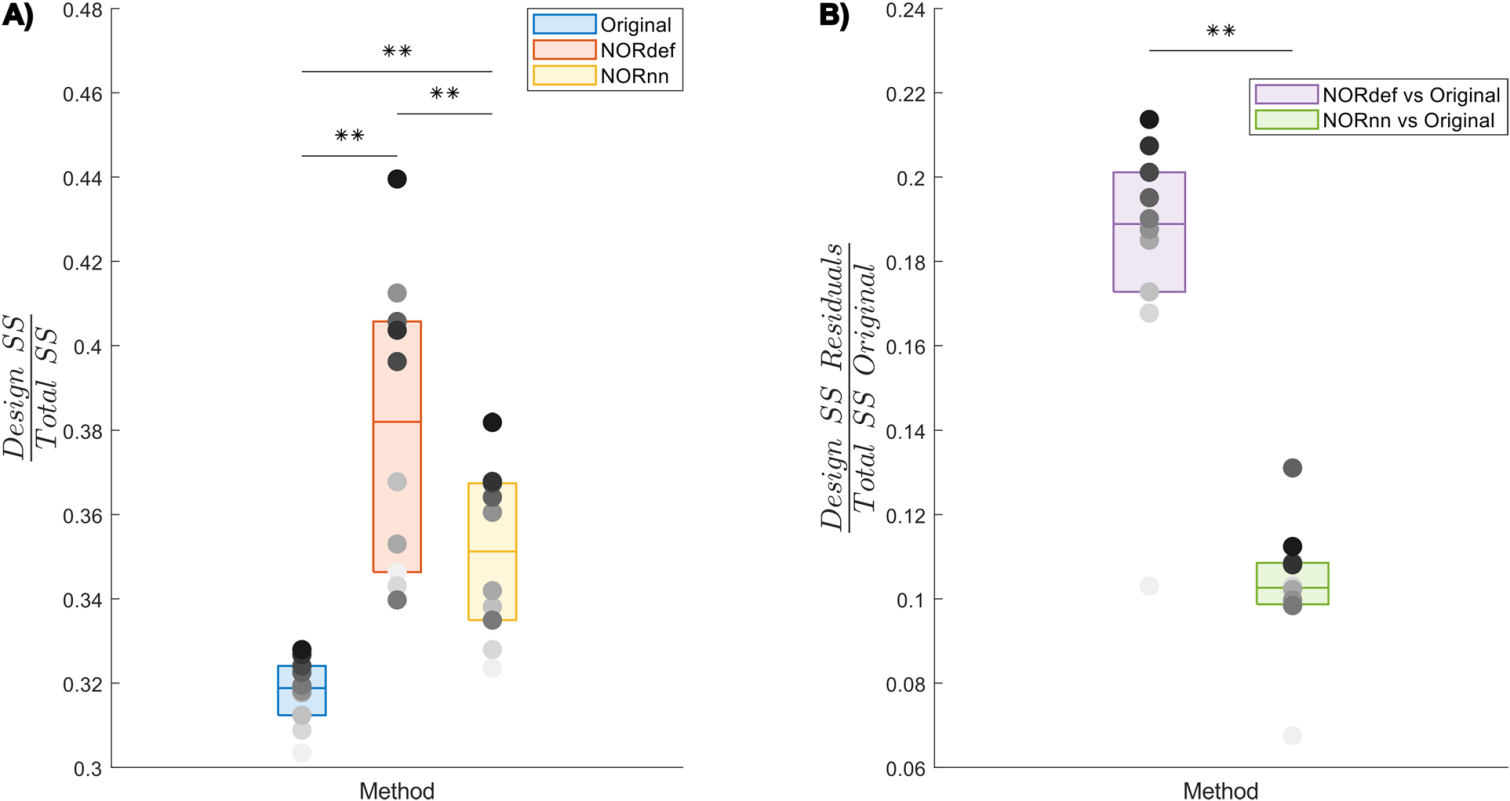
Group analysis of the variance explained by the stimulation design. (A) Box charts show the interquartile percentile range of variance explained by the design in the data across participants. After NORDIC denoising, an increased proportion of the variance is explained by the experimental design. NORnn shows an increase in explained variance compared to the Original data, but a slightly lower increase than NORdef. (B) The proportion of variance explained by the design that is removed from the Original data after NORDIC. NORdef removed a larger proportion of the signal compared to NORnn. Grayscale dots indicate mean values of individual participants ordered according to their mean value in the Original data (participants have the same color in each of the three datasets). ** indicates p < 0.01.

To investigate the nature of the bias introduced by NORDIC further, we quantified the variance explained by the design both in the time series as well as in the portions of the original time series that are not present in either the NORdef or NORnn time series. When computing the variance explained by the design compared to the total variance of the signal (in each respective method dataset—Total SS), denoising resulted in an increasingly higher portion of variance explained by the experimental design (Figs. 7AandS11). This is in line with the increased statistical detection sensitivity afforded by NORDIC denoising (with or without the noise scan -Fig. 2). Interestingly though, after NORDIC, information related to the experimental design was present in the part of the signal that was removed by the denoising procedure. In relation to the total original variance (Total SS Original), the variance explained by the design in the residuals after NORDIC was higher for NORdef compared to NORnn, which is in line with the higher number of principal components that are removed when using NORdef compared to NORnn (Figs. 7BandS12). Permutations indicated that the effects described were significant against an alpha level of 0.05, corrected for multiple comparisons, both for the increase in variance explained by the design in the time series (before and after NORDIC—all p-values were smaller than 0.001) and for the increase in variance explained by the design in the residuals after NORDIC (p < 0.001).

### 3.3 Laminar data

**Fig. 8.**
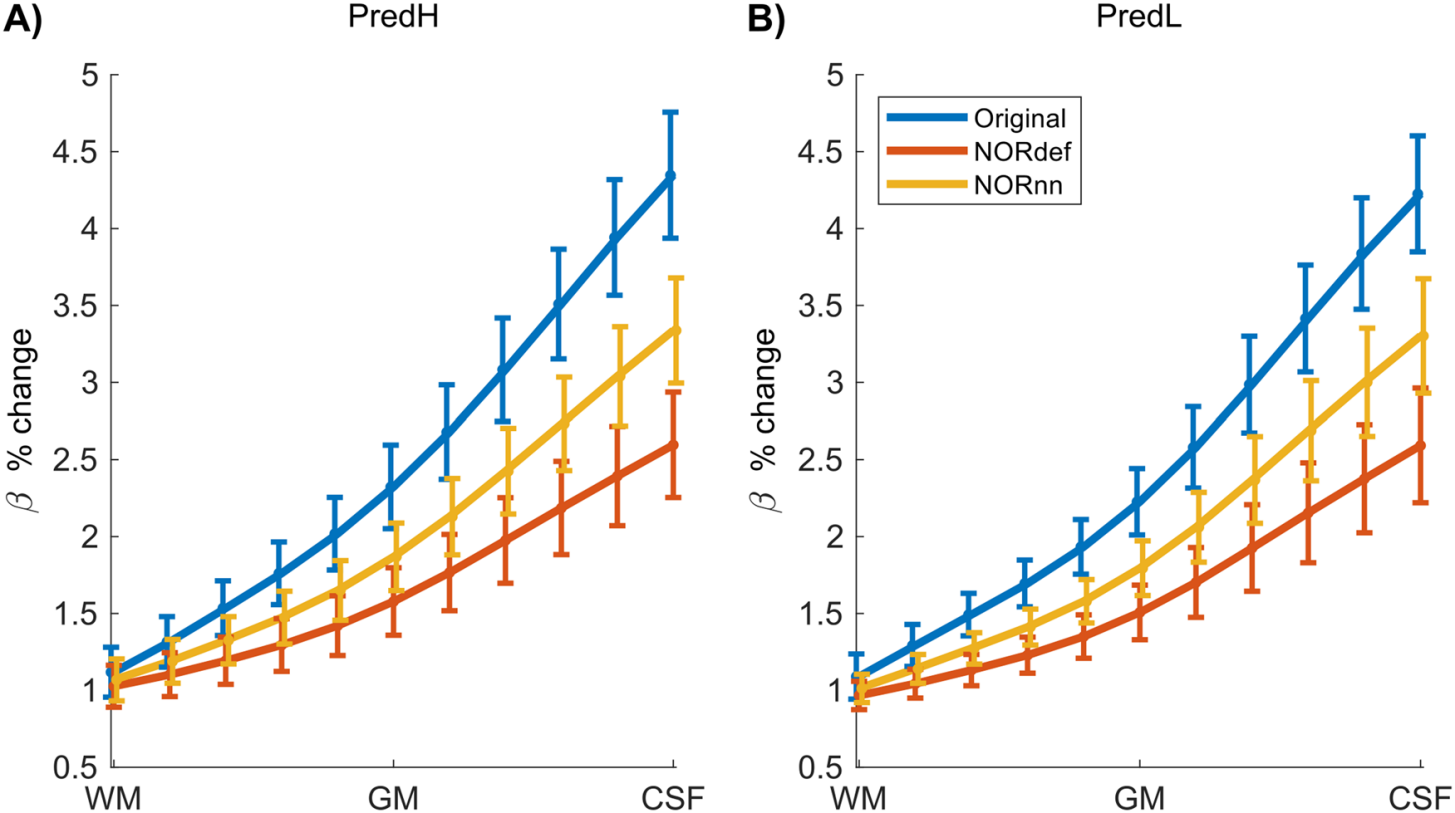
Average effect of NORDIC across depth. Group average laminar plot for the PredH (A) and PredL condition (B). We can easily identify the draining vein effect in both conditions. However, a gradual decrease in slope is visible for NORnn and NORdef, indicating that NORDIC denoising has a differential effect across depths.

Submillimeter data collected with experimental designs presented here are often used to investigate task-related cortical depth dependent changes in functional activity. In preparation for such future studies, we set out to determine the effect that NORDIC has on the laminar profiles of individual conditions. We considered the depth-dependent changes (11 equivolume cortical depths) associated with the PredH and the PredL condition. While in all participants we could observe the expected increase in betas towards the surface in our GE-BOLD data (Heinzle et al., 2016;Menon et al., 1995;Turner, 2002), NORDIC denoising is associated with a clear reduction in percent signal in superficial cortical depths (Figs. 8, andS13andS14for individual subjects).

## 4 Discussion

Functional MRI is an indispensable tool for the investigation of human brain function. However, fMRI data are inherently limited by physiological and thermal noise (Triantafyllou et al., 2005,^2011^). For this reason, in the fMRI community, the development of methods for removing unwanted sources of variance in the data has been a longstanding goal. Denoising techniques in fMRI can be broadly distinguished in those that tackle the removal of structured (physiological) noise and those that instead aim to reduce thermal noise. A technique that has been introduced to deal with thermal noise in particular is NORDIC PCA. NORDIC denoising has been vetted in various brain areas, voxel sizes, experimental designs, acceleration types and their degree, and field strengths (for examples seeDowdle et al., 2022,^2023^;Knudsen et al., 2023;Raimondo et al., 2023;Vizioli et al., 2021). While in fMRI several approaches have been introduced to improve (statistical) detection power of the signals of interest, it is important to note that any denoising approach may affect the temporal or spatial precision of the underlying fMRI signal as well as their bias-variance tradeoff (Kay, 2022). Ideally, denoising techniques should not spatially or temporally blur the data, while also minimizing any bias introduced. In its initial applications to fMRI, NORDIC denoising has been shown to preserve spatial and temporal information as well as not introducing unwanted biases in the data. These applications have focused primarily on visual and motor cortical areas at different magnetic field strengths (3T and 7T) and using different experimental designs as well as contrast mechanisms (Dowdle et al., 2022,^2023^;Knudsen et al., 2023;Raimondo et al., 2023).

Investigating fMRI responses in temporal cortical areas with high spatial resolution (at UHF) is particularly challenging. The location of (primary) cortical areas, in particular, calls for large field-of-view acquisitions (in either transversally or coronally applied slices to ensure bilateral coverage), which requires high in-plane acceleration to reduce distortions in the resulting EPI images. In addition, when using a single transmit coil, as is the case in most applications, inhomogeneities in the radio frequency transmit field result in suboptimal flip angles (Moerel et al., 2021). While ad-hoc solutions can be found (e.g., by limiting the coverage to single hemispheres), high spatial resolution investigations of temporal cortical areas result in lower temporal SNR compared to, for example, visual or motor cortical regions. For these reasons, we evaluated the consequences associated with the use of NORDIC denoising in temporal cortical areas and extend this to a larger number of subjects. We compared two processing strategies to the Original data (i.e., no NORDIC denoising), one dataset using the default settings for fMRI NORDIC (i.e., using magnitude and phase images and a noise threshold estimated using noise scans) and one dataset with a more conservative noise threshold obtained from g-factor estimation.

Our results indicate that NORDIC processing results in increased reliability of the spatial patterns with NORnn (Fig. 3B) and generally an increased reliability in the response estimates (Figs. 5Cand5D, andS7andS8). However, our results suggest that, in auditory cortical regions, NORDIC denoising, in general, is associated with a non-negligible difference in betas, compared to the Original data (Figs. 5AandS5), elicited by our slow event-related design (seeKnudsen et al., 2023; andPfaffenrot & Norris, 2023for similar results in other cortical areas). These effects are reminiscent of regularization approaches in regression as they result in lower estimated regression coefficients (i.e., betas) while reducing their variance. The variance reduction is proportionally larger with respect to the introduced bias, as evidenced by the increased t-statistics (Figs. 5BandS6) and underlies the increased statistical detection sensitivity following NORDIC processing compared to the Original data (Fig. 2- and in agreement with previous studiesDowdle et al., 2023;Vizioli et al., 2021). Importantly, in general, the reduced variance in the NORDIC processed data results in increased spatial consistency when evaluated in a repeated split half analysis in activated areas (Figs. 5Cand5D,S7andS8). All our analyses performed at the level of beta estimates in different temporal cortical regions showed a gradual improvement (e.g., in t-statistics) from NORnn to NORdef (and an associated larger bias in NORdef compared to NORnn), in line with our assumption that NORnn is the more conservative approach. Interestingly, even within a dataset, the deviations from the Original data introduced by NORDIC are not uniform, they are associated with the amount of signal present in the data. That is, voxels with more signal (as measured by the mean of the time series divided by the standard deviation of the time series [tSNR]) show the lowest change in estimated percent signal (Figs. 6andS10).

The difference between the Original data and NORDIC processed data is suggestive of the fact that some signal (associated with the experimental design) has been removed by the approach. We confirmed this by analyzing the portion of the signal from the magnitude images that is removed by NORDIC (computed as the portion of the Original data time series orthogonal to either the NORdef or NORnn time series). While the design explained larger portions of variance in the data after NORDIC processing, the design also explained larger portions of variance in the residuals after NORDIC (Figs. 7,S11andS12). This indicates that, perhaps not surprisingly, NORDIC can remove portions of the signal that in a given sample (i.e., a functional run) are indistinguishable from the noise. These results are in agreement with those presented inFigure 6, indicating larger changes in beta estimates after NORDIC (compared to the Original data) in voxels with lower tSNR (putatively voxels in which the signal and the noise are more confounded—Figs. 6andS10).

As a preliminary analysis, we investigated the difference in laminar profiles between NORDIC and the Original data (Figs. 8, andS13andS14). We show that NORDIC affects cortical depth-dependent profiles with a larger reduction in betas in superficial layers compared to deep layers (more pronounced in NORdef compared to NORnn). This interesting effect may relate to the changes in signal and noise contributions across depths in GE-fMRI. Previous research in one volunteer has shown that this effect is more pronounced around large venous vessels (Pfaffenrot & Norris, 2023), a hypothesis that would deserve further investigation with careful definition of the vessel architecture requiring images with dedicated contrast that were not available in our dataset (see e.g.Moerel et al., 2018;Pfaffenrot & Norris, 2023). The effect that this signal reduction would have on the evaluation of laminar dependent differences between conditions should also be evaluated by designing a sufficiently powered study to establish a ground truth (for differential laminar effects) at the single-subject level and compare Original and NORDIC processed data to this ground truth.

Apart from the changes in betas and improved statistical reliability and spatial similarity, in our data NORDIC had a clear effect on inter-subject variability which increased for spatial correlation metrics, t-statistics, and explained variance (Figs. 3B,3C,5Band7). NORDIC targets the removal of thermal noise that can be distinguished from signal, but the remaining data will still contain physiological noise that is specific to the subject and the specific brain region (and thus the experiment). Compared to the visual cortex, the auditory cortex is more susceptible to physiological noise (Faes et al., 2023;Moerel et al., 2021). Both the magnitude and nature of physiological noise in temporal cortices may hamper the separation of thermal noise from physiological noise. On the one hand, the intrinsic variability of this effect across subjects may contribute to the increased variability we observe—that is, larger increases in t-statistics are observed for subjects in whom physiological and thermal noise are better separable. On the other hand, the separability of physiological noise and thermal noise may also underlie the difference we observe when using the noise scan (NORdef) compared to not using the noise scan (NORnn). In our data, NORnn results in less of a decrease of betas and, also, reduced increases in variability across subjects. Thus, the more conservative threshold resulting from NORnn may protect against the effects introduced by poorer separability of thermal noise and physiological noise. A more detailed investigation of how physiological noise and its separability to thermal noise affects NORDIC processing (both in terms of its potential effect on the definition of the component threshold and thus the bias and the inter-subject variability of this effect) would be of interest for future studies comparing for example visual and auditory responses with block and event-related designs in the same individuals.

It is important to note that we here defined the bias introduced by NORDIC as the reduction in betas that is visible when analyzing the time series after NORDIC compared to the Original data (Figs. 5AandS5). While NORDIC acts on complex data (to ensure a Gaussian distribution of the noise), the percent signal estimates are computed on the magnitude data. The noise distribution in magnitude only data is not Gaussian but Rician (see e.g.Manzano Patron et al., 2024) and can result in a biased estimate of the effects. That is, it is possible that the reduced betas we observe after NORDIC are stemming from a larger bias in the estimates obtained from the Original data induced by the elevated noise floor. While this explanation offers an alternative interpretation of the reduced betas obtained after NORDIC, it is not clear how it can explain the effects we report on the portion of the variance explained by the design in the residuals of the time series after NORDIC (Figs. 7BandS12). This is because any amplitude difference between the original and the NORDIC time series is accounted for in the way we estimate the residuals after NORDIC (i.e., these residuals are not a simple subtraction of the data before and after NORDIC).

Our results have some implications for the use of NORDIC in neuroscientific investigations as well as for future methodological developments of this denoising technique. First, as NORDIC can (in low SNR regimes as ours) remove portions of the signal, it follows that its application on a run-to-run basis may not combine its benefits to the more general practice of averaging. That is, while averaging will preserve all signal portions in the single run data (and with enough runs may render small effects detectable), NORDIC may remove some of these effects in the single runs and make them undetectable even after extensive averaging. Second, any biases introduced by NORDIC are likely related to signal components that, in a given sample (i.e., a run), are indistinguishable from noise. This consideration highlights the need to further investigate the interaction between the experimental design and any bias introduced by NORDIC processing. That is, in our data the effect may have been exacerbated by the low power in our slow event-related stimulus presentation that may confound the response (i.e., the signal) more with the noise in low SNR regimes. While in visual areas event-related designs do not result in a detectable bias after NORDIC (Dowdle et al., 2023), this may relate to the higher SNR of visual areas compared to temporal regions. Finally, it is tempting to speculate that several approaches could be undertaken to abate the bias. Here, we showed that a more conservative threshold for the identification of noisy eigenvalues results in a lower bias (NORnn). Further investigations are warranted in evaluating the effect that other settings (e.g., the patch size) have on the bias (seePfaffenrot & Norris, 2023for a first attempt to quantify the effect of patch size). More sophisticated approaches could be considered to, for example, select principal components for removal only if their relationship with the experimental design is negligible akin to the selection of interesting components when performing independent component analysis for task fMRI (De Martino et al., 2007;McKeown et al., 1998;Moritz et al., 2005;Schmithorst & Brown, 2004). Such an approach would not generalize to resting-state fMRI but could help for task-based functional studies.

Independent of the biases we describe here, NORDIC processing remains an important tool for fMRI investigations, especially when SNR is limited (i.e., when thermal noise is dominant), such as laminar studies or fMRI studies using less sensitive contrast mechanisms such as spin-echo BOLD or non-BOLD contrast mechanisms such as cerebral blood volume-based vascular space occupancy (Huber et al., 2014;Knudsen et al., 2023;Lu, 2008) or blood flow-based contrast mechanisms such as arterial spin labeling (Kashyap et al., 2021). NORDIC can then be used as a complement to techniques that target physiological noise components to improve the usability of these different SNR-starved acquisition approaches. Similarly, NORDIC could be very beneficial in patient studies that cannot rely on long scan times (i.e., extensive averaging) because of practical constraints. In general, though, while any given processing or reconstruction step likely introduces some bias, and while it may be acceptable in some circumstances, it is reasonable to advise NORDIC users to evaluate the amount of bias introduced in their data (by, e.g., plotting percent signal estimates before and after NORDIC) apart from focusing only on the increased (statistical) detectability of the effects.

In conclusion, NORDIC can be added to the family of preprocessing techniques that can be utilized to improve the detection sensitivity and reliability of the responses estimated from the fMRI signal. The improvements NORDIC affords warrant its use in SNR-challenged settings. Following previous reports, also in our data these positive effects were significant. The signal changes we report here, on the other hand, suggest that some care is required when using NORDIC—new applications may have to further characterize the effect of NORDIC to better evaluate the generalizability of its effects.

## Supplementary Material

Supplementary Material

## Data Availability

Preprocessing scripts and Matlab analyses codes are available on Githubhttps://github.com/lonikefaes/auditory_nordic. The anonymized raw data of this study are available and can be downloaded fromdoi:10.18112/openneuro.ds004928.v1.0.0.

## References

[b1] Ahveninen , J. , Chang , W.-T. , Huang , S. , Keil , B. , Kopco , N. , Rossi , S. , Bonmassar , G. , Witzel , T. , & Polimeni , J. R. ( 2016 ). Intracortical depth analyses of frequency-sensitive regions of human auditory cortex using 7T fMRI . NeuroImage , 143 , 116 – 127 . 10.1016/j.neuroimage.2016.09.010 27608603 PMC5124525

[b2] Allen , E. J. , St-Yves , G. , Wu , Y. , Breedlove , J. L. , Prince , J. S. , Dowdle , L. T. , Nau , M. , Caron , B. , Pestilli , F. , Charest , I. , Hutchinson , J. B. , Naselaris , T. , & Kay , K. ( 2022 ). A massive 7T fMRI dataset to bridge cognitive neuroscience and artificial intelligence . Nature Neuroscience , 25 ( 1 ), 116 – 126 . 10.1038/s41593-021-00962-x 34916659

[b3] Behzadi , Y. , Restom , K. , Liau , J. , & Liu , T. T. ( 2007 ). A component based noise correction method (CompCor) for BOLD and perfusion based fMRI . NeuroImage , 37 ( 1 ), 90 – 101 . 10.1016/j.neuroimage.2007.04.042 17560126 PMC2214855

[b4] Berlot , E. , Formisano , E. , & De Martino , F. ( 2018 ). Mapping frequency-specific tone predictions in the human auditory cortex at high spatial resolution . The Journal of Neuroscience , 38 ( 21 ), 4934 – 4942 . 10.1523/JNEUROSCI.2205-17.2018 29712781 PMC6596130

[b5] Cordero-Grande , L. , Christiaens , D. , Hutter , J. , Price , A. N. , & Hajnal , J. V. ( 2019 ). Complex diffusion-weighted image estimation via matrix recovery under general noise models . NeuroImage , 200 , 391 – 404 . 10.1016/j.neuroimage.2019.06.039 31226495 PMC6711461

[b6] De Martino , F. , Gentile , F. , Esposito , F. , Balsi , M. , Di Salle , F. , Goebel , R. , & Formisano , E. ( 2007 ). Classification of fMRI independent components using IC-fingerprints and support vector machine classifiers . NeuroImage , 34 ( 1 ), 177 – 194 . 10.1016/j.neuroimage.2006.08.041 17070708

[b7] De Martino , F. , Moerel , M. , Ugurbil , K. , Goebel , R. , Yacoub , E. , & Formisano , E. ( 2015 ). Frequency preference and attention effects across cortical depths in the human primary auditory cortex . Proceedings of the National Academy of Sciences , 112 ( 52 ), 16036 – 16041 . 10.1073/pnas.1507552112 PMC4702984 26668397

[b8] De Martino , F. , Yacoub , E. , Kemper , V. , Moerel , M. , Uludağ , K. , De Weerd , P. , Ugurbil , K. , Goebel , R. , & Formisano , E. ( 2018 ). The impact of ultra-high field MRI on cognitive and computational neuroimaging . NeuroImage , 168 , 366 – 382 . 10.1016/j.neuroimage.2017.03.060 28396293

[b9] Dowdle , L. , Ghose , G. , Moeller , S. , Ugurbil , K. , Yacoub , E. , & Vizioli , L. ( 2022 ). Task demands differentiate regional depth-dependent activity profiles within the ventral visual pathway . BioRxiv . 10.1101/2022.12.03.518973

[b10] Dowdle , L. , Vizioli , L. , Moeller , S. , Akçakaya , M. , Olman , C. , Ghose , G. , Yacoub , E. , & Uğurbil , K. ( 2023 ). Evaluating increases in sensitivity from NORDIC for diverse fMRI acquisition strategies . NeuroImage , 270 , 119949 . 10.1016/j.neuroimage.2023.119949 36804422 PMC10234612

[b11] Dumoulin , S. O. , Fracasso , A. , van der Zwaag , W. , Siero , J. C. W. , & Petridou , N. ( 2018 ). Ultra-high field MRI_ Advancing systems neuroscience towards mesoscopic human brain function . NeuroImage , 168 , 345 – 357 . 10.1016/j.neuroimage.2017.01.028 28093360

[b12] Faes , L. K. , De Martino , F. , & Huber , L. ( 2023 ). Cerebral blood volume sensitive layer-fMRI in the human auditory cortex at 7T: Challenges and capabilities . PLoS One , 18 ( 2 ), e0280855 . 10.1371/journal.pone.0280855 36758009 PMC9910709

[b13] Fernandes , F. F. , Olesen , J. L. , Jespersen , S. N. , & Shemesh , N. ( 2023 ). MP-PCA denoising of fMRI time-series data can lead to artificial activation “spreading.” NeuroImage , 273 , 120118 . 10.1016/j.neuroimage.2023.120118 37062372

[b14] Formisano , E. , Kim , D.-S. , Di Salle , F. , van de Moortele , P.-F. , Ugurbil , K. , & Goebel , R. ( 2003 ). Mirror-symmetric tonotopic maps in human primary auditory cortex . Neuron , 40 ( 4 ), 859 – 869 . 10.1016/S0896-6273(03)00669-X 14622588

[b15] Formisano , E. , & Kriegeskorte , N. ( 2012 ). Seeing patterns through the hemodynamic veil—The future of pattern-information fMRI . NeuroImage , 62 ( 2 ), 1249 – 1256 . 10.1016/j.neuroimage.2012.02.078 22421051

[b16] Gau , R. , Bazin , P.-L. , Trampel , R. , Turner , R. , & Noppeney , U. ( 2020 ). Resolving multisensory and attentional influences across cortical depth in sensory cortices . eLife , 9 , e46856 . 10.7554/eLife.46856 31913119 PMC6984812

[b17] Glover , G. H. , Li , T.-Q. , & Ress , D. ( 2000 ). Image-based method for retrospective correction of physiological motion effects in fMRI: RETROICOR . Magnetic Resonance in Medicine , 44 ( 1 ), 162 – 167 . 10.1002/1522-2594(200007)44:1<162::AID-MRM23>3.0.CO;2-E 10893535

[b18] Gonzalez-Castillo , J. , Panwar , P. , Buchanan , L. C. , Caballero-Gaudes , C. , Handwerker , D. A. , Jangraw , D. C. , Zachariou , V. , Inati , S. , Roopchansingh , V. , Derbyshire , J. A. , & Bandettini , P. A. ( 2016 ). Evaluation of multi-echo ICA denoising for task based fMRI studies: Block designs, rapid event-related designs, and cardiac-gated fMRI . NeuroImage , 141 , 452 – 468 . 10.1016/j.neuroimage.2016.07.049 27475290 PMC5026969

[b19] Griffanti , L. , Salimi-Khorshidi , G. , Beckmann , C. F. , Auerbach , E. J. , Douaud , G. , Sexton , C. E. , Zsoldos , E. , Ebmeier , K. P. , Filippini , N. , Mackay , C. E. , Moeller , S. , Xu , J. , Yacoub , E. , Baselli , G. , Ugurbil , K. , Miller , K. L. , & Smith , S. M. ( 2014 ). ICA-based artefact removal and accelerated fMRI acquisition for improved resting state network imaging . NeuroImage , 95 , 232 – 247 . 10.1016/j.neuroimage.2014.03.034 24657355 PMC4154346

[b20] Heinzle , J. , Koopmans , P. J. , den Ouden , H. E. M. , Raman , S. , & Stephan , K. E. ( 2016 ). A hemodynamic model for layered BOLD signals . NeuroImage , 125 , 556 – 570 . 10.1016/j.neuroimage.2015.10.025 26484827

[b21] Heynckes , M. , Lage-Castellanos , A. , De Weerd , P. , Formisano , E. , & De Martino , F. ( 2023 ). Layer-specific correlates of detected and undetected auditory targets during attention . Current Research in Neurobiology , 4 , 100075 . 10.1016/j.crneur.2023.100075 36755988 PMC9900365

[b22] Hu , X. , Le , T. H. , Parrish , T. , & Erhard , P. ( 1995 ). Retrospective estimation and correction of physiological fluctuation in functional MRI . Magnetic Resonance in Medicine , 34 ( 2 ), 201 – 212 . 10.1002/mrm.1910340211 7476079

[b23] Huber , L. , Goense , J. , Kennerley , A. J. , Trampel , R. , Guidi , M. , Reimer , E. , Ivanov , D. , Neef , N. , Gauthier , C. J. , Turner , R. , & Möller , H. E. ( 2015 ). Cortical lamina-dependent blood volume changes in human brain at 7 T . NeuroImage , 107 , 23 – 33 . 10.1016/j.neuroimage.2014.11.046 25479018

[b24] Huber , L. , Ivanov , D. , Krieger , S. N. , Streicher , M. N. , Mildner , T. , Poser , B. A. , Möller , H. E. , & Turner , R. ( 2014 ). Slab-selective, BOLD-corrected VASO at 7 Tesla provides measures of cerebral blood volume reactivity with high signal-to-noise ratio: SS-SI-VASO measures changes of CBV in brain . Magnetic Resonance in Medicine , 72 ( 1 ), 137 – 148 . 10.1002/mrm.24916 23963641

[b25] Huber , L. , Poser , B. A. , Bandettini , P. A. , Arora , K. , Wagstyl , K. , Cho , S. , Goense , J. , Nothnagel , N. , Morgan , A. T. , van den Hurk , J. , Müller , A. K. , Reynolds , R. C. , Glen , D. R. , Goebel , R. , & Gulban , O. F. ( 2021 ). LayNii: A software suite for layer-fMRI . NeuroImage , 237 , 118091 . 10.1016/j.neuroimage.2021.118091 33991698 PMC7615890

[b26] Kashyap , S. , Ivanov , D. , Havlicek , M. , Huber , L. , Poser , B. A. , & Uludağ , K. ( 2021 ). Sub-millimetre resolution laminar fMRI using arterial spin labelling in humans at 7 T . PLoS One , 16 ( 4 ), e0250504 . 10.1371/journal.pone.0250504 33901230 PMC8075193

[b27] Kay , K. N. ( 2022 ). The risk of bias in denoising methods: Examples from neuroimaging . PLoS One , 17 ( 7 ), e0270895 . 10.1371/journal.pone.0270895 35776751 PMC9249232

[b28] Kay , K. N. , Rokem , A. , Winawer , J. , Dougherty , R. F. , & Wandell , B. A. ( 2013 ). GLMdenoise: A fast, automated technique for denoising task-based fMRI data . Frontiers in Neuroscience , 7 , 247 . 10.3389/fnins.2013.00247 24381539 PMC3865440

[b29] Kiebel , S. J. , Goebel , R. , & Friston , K. J. ( 2000 ). Anatomically informed basis functions . NeuroImage , 11 ( 6 ), 656 – 667 . 10.1006/nimg.1999.0542 10860794

[b200] Kim , J. J. , Crespo-Facorro , B. , Andreasen , N. C. , O’Leary , D. S. , Zhang , B. , Harris , G. , & Magnotta , V. A. ( 2000 ). An MRI-based parcellation method for the temporal lobe . Neuroimage , 11 ( 4 ), 271 – 288 . 10.1006/nimg.2000.0543 10725184

[b30] Knudsen , L. , Bailey , C. J. , Blicher , J. U. , Yang , Y. , Zhang , P. , & Lund , T. E. ( 2023 ). Improved sensitivity and microvascular weighting of 3T laminar fMRI with GE-BOLD using NORDIC and phase regression . NeuroImage , 271 , 120011 . 10.1016/j.neuroimage.2023.120011 36914107

[b31] Kok , P. , Bains , L. J. , van Mourik , T. , Norris , D. G. , & de Lange , F. P. ( 2016 ). Selective activation of the deep layers of the human primary visual cortex by top-down feedback . Current Biology , 26 ( 3 ), 371 – 376 . 10.1016/j.cub.2015.12.038 26832438

[b32] Lawrence , S. J. , Norris , D. G. , & de Lange , F. P. ( 2019 ). Dissociable laminar profiles of concurrent bottom-up and top-down modulation in the human visual cortex . eLife , 8 , e44422 . 10.7554/eLife.44422 31063127 PMC6538372

[b33] Lu , H. ( 2008 ). Magnetization “reset” for non-steady-state blood spins in vascular-space-occupancy (VASO) fMRI . Proceedings of 16th Annual Meeting of ISMRM . Toronto, Canada , 406 . https://cds.ismrm.org/protected/08MProceedings/PDFfiles/00406.pdf

[b34] Ma , X. , Uğurbil , K. , & Wu , X. ( 2020 ). Denoise magnitude diffusion magnetic resonance images via variance-stabilizing transformation and optimal singular-value manipulation . NeuroImage , 215 , 116852 . 10.1016/j.neuroimage.2020.116852 32305566 PMC7292796

[b35] Manzano Patron , J. P. , Moeller , S. , Andersson , J. L. R. , Ugurbil , K. , Yacoub , E. , & Sotiropoulos , S. N. ( 2024 ). Denoising diffusion MRI: Considerations and implications for analysis . Imaging Neuroscience , 2 , 1 – 29 . 10.1162/imag_a_00060 PMC12224456 40800437

[b36] Marques , J. P. , Kober , T. , Krueger , G. , van der Zwaag , W. , Van de Moortele , P.-F. , & Gruetter , R. ( 2010 ). MP2RAGE, a self bias-field corrected sequence for improved segmentation and T1-mapping at high field . NeuroImage , 49 ( 2 ), 1271 – 1281 . 10.1016/j.neuroimage.2009.10.002 19819338

[b37] McKeown , M. J. , Makeig , S. , Brown , G. G. , Jung , T.-P. , Kindermann , S. S. , Bell , A. J. , & Sejnowski , T. J. ( 1998 ). Analysis of fMRI data by blind separation into independent spatial components . Human Brain Mapping , 6 ( 3 ), 160 – 188 . 10.1002/(SICI)1097-0193(1998)6:3<160::AID-HBM5>3.0.CO;2-1 9673671 PMC6873377

[b38] Menon , R. S. , Ogawa , S. , Hu , X. , Strupp , J. P. , Anderson , P. , & Ugurbil , K. ( 1995 ). BOLD based functional MRI at 4 Tesla includes a capillary bed contribution: Echo-planar imaging correlates with previous optical imaging using intrinsic signals . Magnetic Resonance in Medicine , 33 , 453 – 459 . 10.1002/mrm.1910330323 7760717

[b39] Michon , K. J. , Khammash , D. , Simmonite , M. , Hamlin , A. M. , & Polk , T. A. ( 2022 ). Person-specific and precision neuroimaging: Current methods and future directions . NeuroImage , 263 , 119589 . 10.1016/j.neuroimage.2022.119589 36030062

[b40] Moeller , S. , Pisharady , P. K. , Ramanna , S. , Lenglet , C. , Wu , X. , Dowdle , L. , Yacoub , E. , Uğurbil , K. , & Akçakaya , M. ( 2021 ). NOise reduction with DIstribution Corrected (NORDIC) PCA in dMRI with complex-valued parameter-free locally low-rank processing . NeuroImage , 226 , 117539 . 10.1016/j.neuroimage.2020.117539 33186723 PMC7881933

[b41] Moeller , S. , Yacoub , E. , Olman , C. A. , Auerbach , E. , Strupp , J. , Harel , N. , & Uğurbil , K. ( 2010 ). Multiband multislice GE-EPI at 7 tesla, with 16-fold acceleration using partial parallel imaging with application to high spatial and temporal whole-brain fMRI . Magnetic Resonance in Medicine , 63 ( 5 ), 1144 – 1153 . 10.1002/mrm.22361 20432285 PMC2906244

[b42] Moerel , M. , De Martino , F. , & Formisano , E. ( 2014 ). An anatomical and functional topography of human auditory cortical areas . Frontiers in Neuroscience , 8 , 225 . 10.3389/fnins.2014.00225 25120426 PMC4114190

[b43] Moerel , M. , De Martino , F. , Kemper , V. G. , Schmitter , S. , Vu , A. T. , Uğurbil , K. , Formisano , E. , & Yacoub , E. ( 2018 ). Sensitivity and specificity considerations for fMRI encoding, decoding, and mapping of auditory cortex at ultra-high field . NeuroImage , 164 , 18 – 31 . 10.1016/j.neuroimage.2017.03.063 28373123 PMC5623610

[b44] Moerel , M. , Yacoub , E. , Gulban , O. F. , Lage-Castellanos , A. , & De Martino , F. ( 2021 ). Using high spatial resolution fMRI to understand representation in the auditory network . Progress in Neurobiology , 207 , 101887 . 10.1016/j.pneurobio.2020.101887 32745500 PMC7854960

[b45] Moritz , C. H. , Carew , J. D. , McMillan , A. B. , & Meyerand , M. E. ( 2005 ). Independent component analysis applied to self-paced functional MR imaging paradigms . NeuroImage , 25 ( 1 ), 181 – 192 . 10.1016/j.neuroimage.2004.11.009 15734354

[b46] Ogawa , S. , Tank , D. W. , Menon , R. , Ellermann , J. M. , Kim , S. G. , Merkle , H. , & Ugurbil , K. ( 1992 ). Intrinsic signal changes accompanying sensory stimulation: Functional brain mapping with magnetic resonance imaging . Proceedings of the National Academy of Sciences , 89 ( 13 ), 5951 – 5955 . 10.1073/pnas.89.13.5951 PMC402116 1631079

[b47] Olman , C. A. , Harel , N. , Feinberg , D. A. , He , S. , Zhang , P. , Ugurbil , K. , & Yacoub , E. ( 2012 ). Layer-specific fMRI reflects different neuronal computations at different depths in human V1 . PLoS One , 7 ( 3 ), e32536 . 10.1371/journal.pone.0032536 22448223 PMC3308958

[b48] Pfaffenrot , V. , & Norris , D. ( 2023 ). Validating NORDIC denoising on high-resolution fMRI data at 7 T . In Proceedings of 32nd Annual Meeting of ISMRM, Toronto, Canada . Abstract 2719. 10.58530/2022/2190

[b49] Poldrack , R. A. , Baker , C. I. , Durnez , J. , Gorgolewski , K. J. , Matthews , P. M. , Munafò , M. R. , Nichols , T. E. , Poline , J.-B. , Vul , E. , & Yarkoni , T. ( 2017 ). Scanning the horizon: Towards transparent and reproducible neuroimaging research . Nature Reviews Neuroscience , 18 ( 2 ), 115 – 126 . 10.1038/nrn.2016.167 28053326 PMC6910649

[b50] Polimeni , J. R. , Renvall , V. , Zaretskaya , N. , & Fischl , B. ( 2018 ). Analysis strategies for high-resolution UHF-fMRI data . NeuroImage , 168 , 296 – 320 . 10.1016/j.neuroimage.2017.04.053 28461062 PMC5664177

[b51] Pruessmann , K. P. , Weiger , M. , Scheidegger , M. B. , & Boesiger , P. ( 1999 ). SENSE: Sensitivity encoding for fast MRI . Magnetic Resonance in Medicine , 42 ( 5 ), 952 – 962 . 10.1002/(SICI)1522-2594(199911)42:5<952::AID-MRM16>3.0.CO;2-S 10542355

[b52] Pruim , R. H. R. , Mennes , M. , van Rooij , D. , Llera , A. , Buitelaar , J. K. , & Beckmann , C. F. ( 2015 ). ICA-AROMA: A robust ICA-based strategy for removing motion artifacts from fMRI data . NeuroImage , 112 , 267 – 277 . 10.1016/j.neuroimage.2015.02.064 25770991

[b53] Raimondo , L. , Priovoulos , N. , Passarinho , C. , Heij , J. , Knapen , T. , Dumoulin , S. O. , Siero , J. C. W. , & van der Zwaag , W. ( 2023 ). Robust high spatio-temporal line-scanning fMRI in humans at 7T using multi-echo readouts, denoising and prospective motion correction . Journal of Neuroscience Methods , 384 , 109746 . 10.1016/j.jneumeth.2022.109746 36403778

[b54] Salimi-Khorshidi , G. , Douaud , G. , Beckmann , C. F. , Glasser , M. F. , Griffanti , L. , & Smith , S. M. ( 2014 ). Automatic denoising of functional MRI data: Combining independent component analysis and hierarchical fusion of classifiers . NeuroImage , 90 , 449 – 468 . 10.1016/j.neuroimage.2013.11.046 24389422 PMC4019210

[b55] Schmithorst , V. J. , & Brown , R. D. ( 2004 ). Empirical validation of the triple-code model of numerical processing for complex math operations using functional MRI and group Independent Component Analysis of the mental addition and subtraction of fractions . NeuroImage , 22 ( 3 ), 1414 – 1420 . 10.1016/j.neuroimage.2004.03.021 15219612

[b56] Setsompop , K. , Cohen-Adad , J. , Gagoski , B. A. , Raij , T. , Yendiki , A. , Keil , B. , Wedeen , V. J. , & Wald , L. L. ( 2012 ). Improving diffusion MRI using simultaneous multi-slice echo planar imaging . NeuroImage , 63 ( 1 ), 569 – 580 . 10.1016/j.neuroimage.2012.06.033 22732564 PMC3429710

[b57] Steel , A. , Garcia , B. D. , Silson , E. H. , & Robertson , C. E. ( 2022 ). Evaluating the efficacy of multi-echo ICA denoising on model-based fMRI . NeuroImage , 264 , 119723 . 10.1016/j.neuroimage.2022.119723 36328274

[b58] Triantafyllou , C. , Hoge , R. D. , Krueger , G. , Wiggins , C. J. , Potthast , A. , Wiggins , G. C. , & Wald , L. L. ( 2005 ). Comparison of physiological noise at 1.5 T, 3 T and 7 T and optimization of fMRI acquisition parameters . NeuroImage , 26 ( 1 ), 243 – 250 . 10.1016/j.neuroimage.2005.01.007 15862224

[b59] Triantafyllou , C. , Polimeni , J. R. , & Wald , L. L. ( 2011 ). Physiological noise and signal-to-noise ratio in fMRI with multi-channel array coils . NeuroImage , 55 ( 2 ), 597 – 606 . 10.1016/j.neuroimage.2010.11.084 21167946 PMC3039683

[b60] Turner , R. ( 2002 ). How much cortex can a vein drain? Downstream dilution of activation-related cerebral blood oxygenation changes . NeuroImage , 16 ( 4 ), 1062 – 1067 . 10.1006/nimg.2002.1082 12202093

[b61] Turner , R. , & Geyer , S. ( 2014 ). Comparing like with like: The power of knowing where you are . Brain Connectivity , 4 ( 7 ), 547 – 557 . 10.1089/brain.2014.0261 24999746 PMC4146387

[b62] Uğurbil , K. ( 2018 ). Imaging at ultrahigh magnetic fields: History, challenges, and solutions . NeuroImage , 168 , 7 – 32 . 10.1016/j.neuroimage.2017.07.007 28698108 PMC5758441

[b63] Valente , G. , Castellanos , A. L. , Hausfeld , L. , De Martino , F. , & Formisano , E. ( 2021 ). Cross-validation and permutations in MVPA: Validity of permutation strategies and power of cross-validation schemes . NeuroImage , 238 , 118145 . 10.1016/j.neuroimage.2021.118145 33961999

[b64] Vaughan , J. T. , Garwood , M. , Collins , C. M. , Liu , W. , DelaBarre , L. , Adriany , G. , Andersen , P. , Merkle , H. , Goebel , R. , Smith , M. B. , & Ugurbil , K. ( 2001 ). 7T vs. 4T: RF power, homogeneity, and signal-to-noise comparison in head images . Magnetic Resonance in Medicine , 46 ( 1 ), 24 – 30 . 10.1002/mrm.1156 11443707

[b65] Veraart , J. , Novikov , D. S. , Christiaens , D. , Ades-aron , B. , Sijbers , J. , & Fieremans , E. ( 2016 ). Denoising of diffusion MRI using random matrix theory . NeuroImage , 142 , 394 – 406 . 10.1016/j.neuroimage.2016.08.016 27523449 PMC5159209

[b66] Vizioli , L. , Moeller , S. , Dowdle , L. , Akçakaya , M. , De Martino , F. , Yacoub , E. , & Uğurbil , K. ( 2021 ). Lowering the thermal noise barrier in functional brain mapping with magnetic resonance imaging . Nature Communications , 12 ( 1 ), 5181 . 10.1038/s41467-021-25431-8 PMC8405721 34462435

[b67] Yacoub , E. , Harel , N. , & Uğurbil , K. ( 2008 ). High-field fMRI unveils orientation columns in humans . Proceedings of the National Academy of Sciences , 105 ( 30 ), 10607 – 10612 . 10.1073/pnas.0804110105 PMC2492463 18641121

[b68] Yacoub , E. , Shmuel , A. , Pfeuffer , J. , Van De Moortele , P.-F. , Adriany , G. , Andersen , P. , Vaughan , J. T. , Merkle , H. , Ugurbil , K. , & Hu , X. ( 2001 ). Imaging brain function in humans at 7 Tesla . Magnetic Resonance in Medicine , 45 ( 4 ), 588 – 594 . 10.1002/mrm.1080 11283986

[b69] Yushkevich , P. A. , Piven , J. , Hazlett , H. C. , Smith , R. G. , Ho , S. , Gee , J. C. , & Gerig , G. ( 2006 ). User-guided 3D active contour segmentation of anatomical structures: Significantly improved efficiency and reliability . NeuroImage , 31 ( 3 ), 1116 – 1128 . 10.1016/j.neuroimage.2006.01.015 16545965

[b70] Zimmermann , J. , Goebel , R. , De Martino , F. , van de Moortele , P.-F. , Feinberg , D. , Adriany , G. , Chaimow , D. , Shmuel , A. , Uğurbil , K. , & Yacoub , E. ( 2011 ). Mapping the organization of axis of motion selective features in human area MT using high-field fMRI . PLoS One , 6 ( 12 ), e28716 . 10.1371/journal.pone.0028716 22163328 PMC3233606

