# Supplementary material for: Evaluating the effect of denoising submillimeter auditory fMRI data with NORDIC
Source: Imaging Neurosci (Camb). 2024 Aug 14;2:imag-2-00270. doi: 10.1162/imag_a_00270 (PMC11726685; doi:10.1162/imag_a_00270)
Supplement: Supplementary Material [file imag_a_00270-supp.pdf]

Supplementary Material

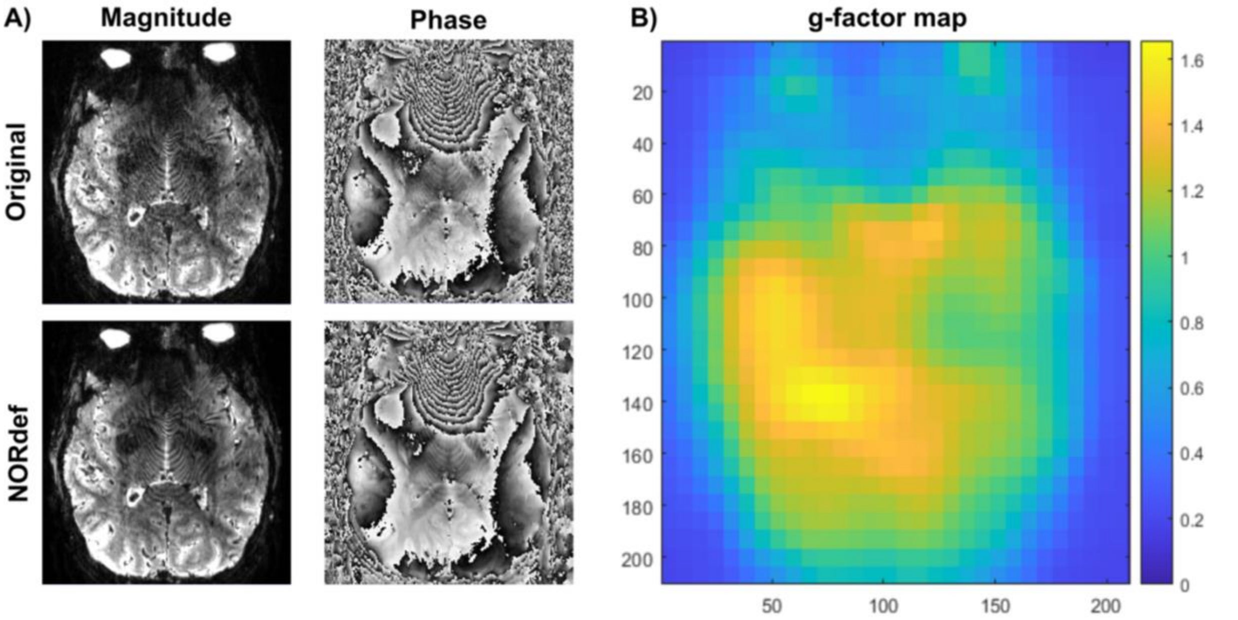

Figure S1. Processing with NORDIC. A) Magnitude and phase images before and after the default NORDIC processing. The magnitude images display less salt and pepper noise after NORDIC processing. B) A map of the g-factor of one exemplary slice.

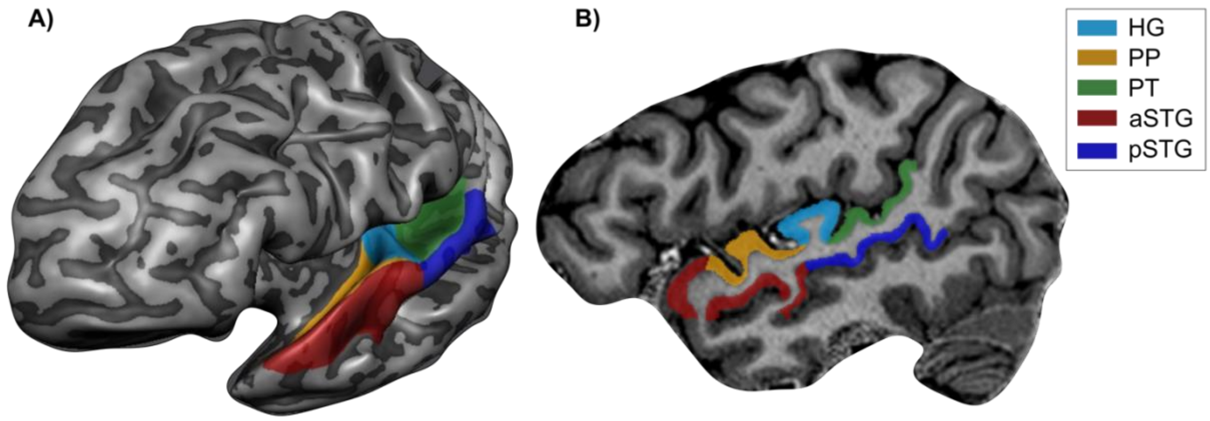

Figure S2. Exemplary illustration of regions of interest. A) On a partially inflated left hemisphere we show the regions of interest that were manually drawn on the mid-gray matter surface, based on microanatomical landmarks described in Kim et al., (2000). B) These ROIs were projected back into the volume and intersected with a gray matter mask. HG = Heschl's Gyrus, PP = Planum Polare, PT = Planum Temporal, aSTG = anterior Superior Temporal Gyrus, pSTG = posterior Superior Temporal Gyrus.

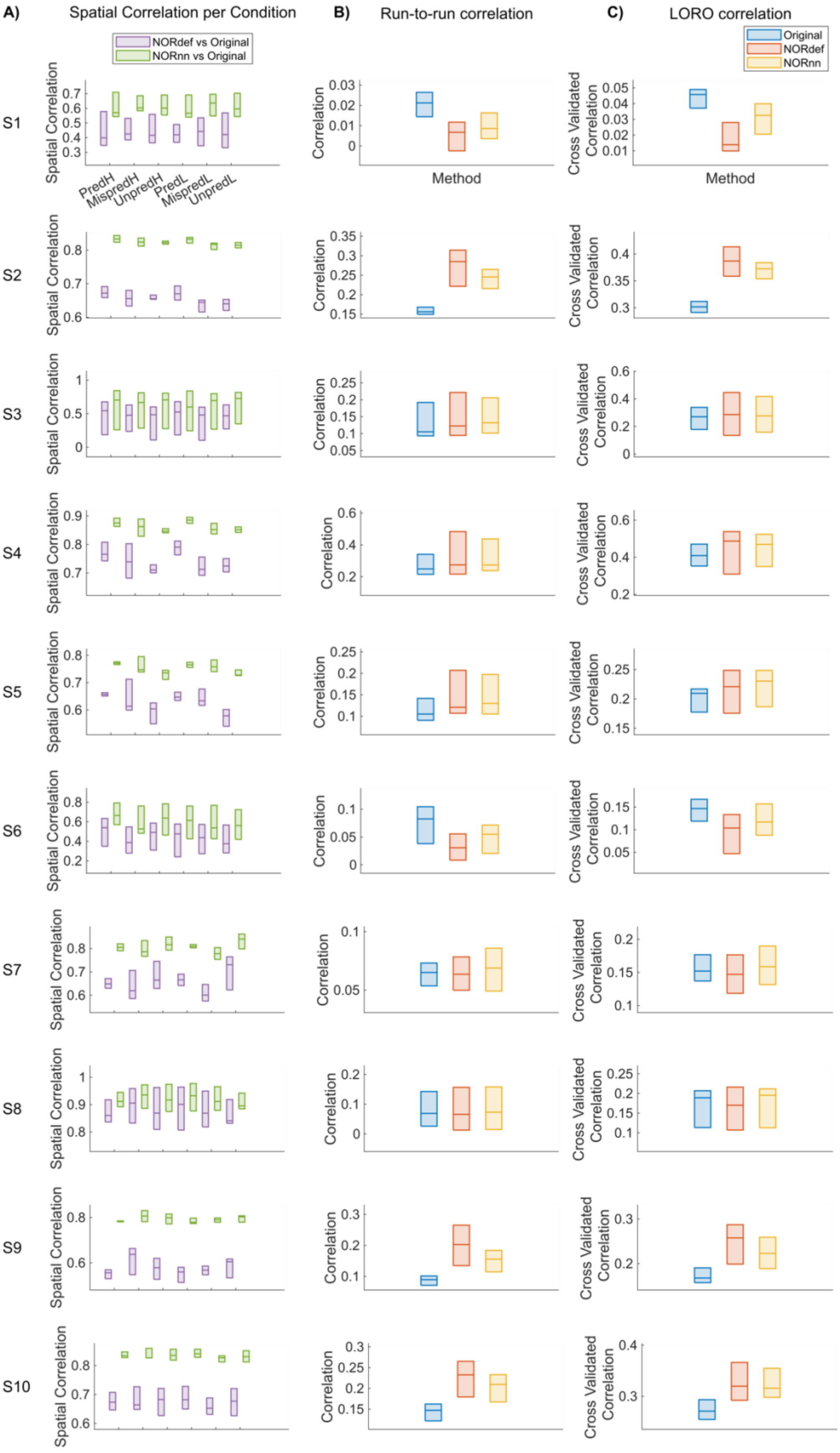
A)
Spatial Correlation per Condition
NORdef vs Original
NORnn vs Original
B)
Run-to-run correlation
C)
LORO correlation
Original
NORdef
NORnn
S1
S2
S3
S4
S5
S6
S7
S8
S9
S10
Spatial Correlation
Correlation
Cross Validated Correlation
Method
PredH
MispredH
UnpredH
PredL
MispredL
UnpredL

Figure S3. Correlation analysis for each individual subject. This figure shows the individual subject plots that are combined in the group analysis in Figure 3. Box charts display the median and interquartile ranges across runs. A) Spatial correlations of beta maps for each condition. The correlation values between NORnn and the Original dataset are higher than the correlation values between NORdef and the Original data, indicating that noise removal in NORnn is more conservative than in the NORdef dataset. B) Run-to-run pairwise correlations computed per dataset for the PredH condition. There seems to be a trend that beta estimates across runs become more similar in both denoised datasets (see Figure 3 for group analysis and statistical testing). C) Cross-validated correlation of one run to the average of n-1 runs of the Original data for the PredH condition.

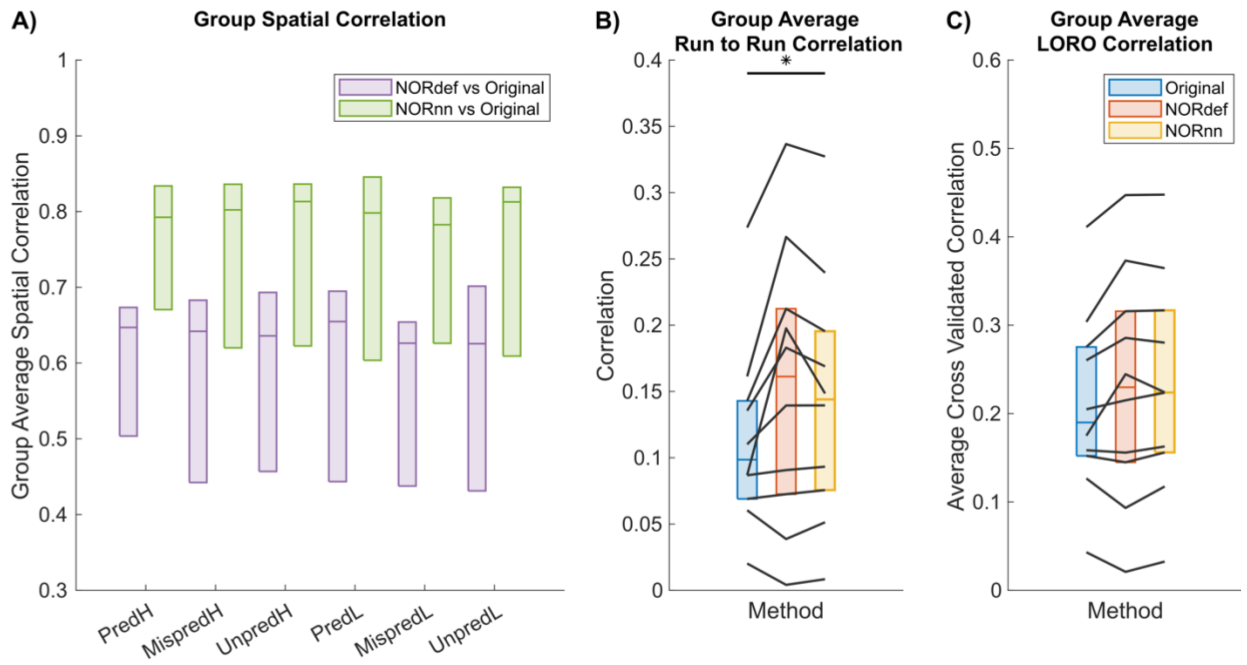

Figures S4. Group Average Correlation Analyses with single subject lines. Same as Figure 3, but lines (instead of dots) indicate single subject data in parts B and C. A) NORnn is more similar to the Original data than NORdef. However, across conditions, there is no evidence of a difference between correlation values between the NORDIC denoised datasets and the Original data (two-way repeated measures ANOVA showed no main effect of condition). This indicates that the number of repetitions of each condition do not affect the effect of NORDIC denoising. B) The run-to-run stability of beta estimates increases significantly with the use of NORnn, i.e. across subjects, beta estimates are more similar across runs after NORnn. C) Average cross validated correlation values of single runs to the average of the Original data for the PredH condition. Lines indicate mean correlation values of individual participants. * indicates $p<0.05$.

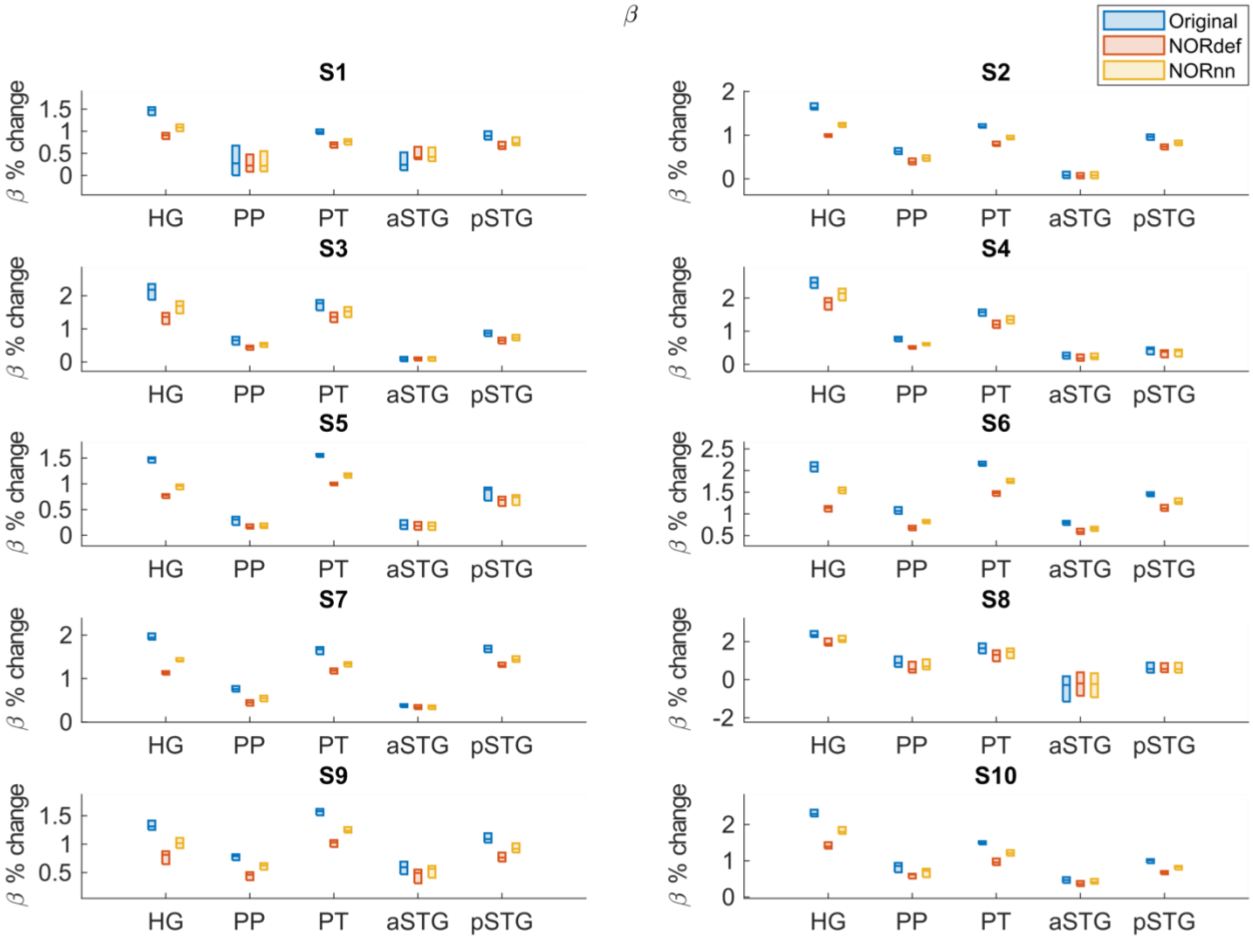

Figure S5. Beta values calculated in percent signal change. This figure illustrates the data from all individual participants, from the group analysis displayed in Figure 5A. In each ROI where there is signal present in the Original dataset, we observed a reduction in beta values after denoising. This reduction was lower in NORnn compared to NORdef. Note that the y-axes differ across participants to appreciate the differences of the three methods for each individual participant.

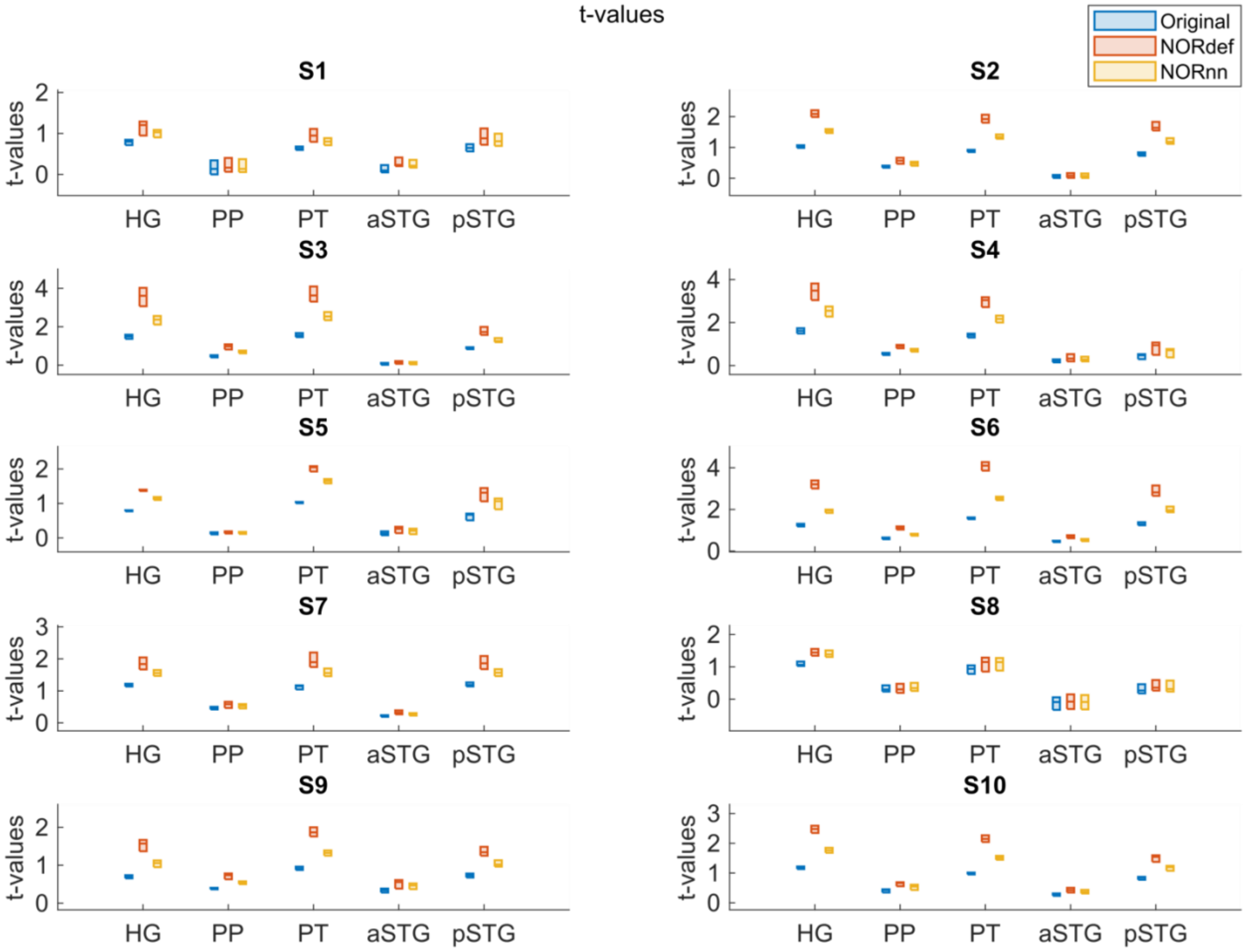

Figure S6. T-values. This figure illustrates the data from all individual participants, from the group analysis displayed in Figure 5B. T-statistics are increased after denoising, which was most pronounced in the NORdef dataset. Note that the y-axes differ across participants to appreciate the differences of the three methods for each individual participant.

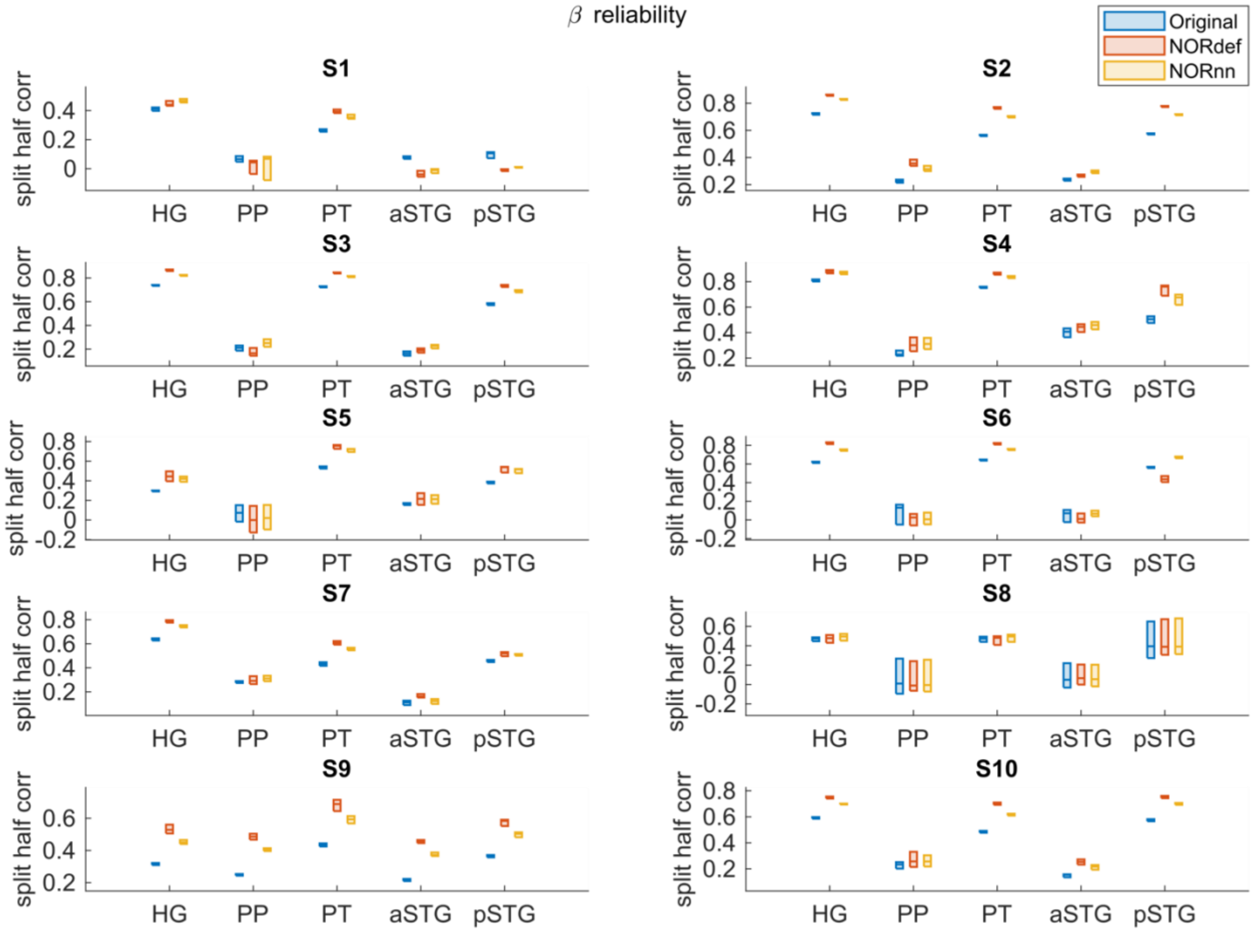

Figure S7. Beta-value reliability. This figure illustrates the data from all individual participants, from the group analysis displayed in Figure 5C. Split half correlations were calculated to estimate the stability of beta responses. This revealed that beta values are generally more stably estimated after denoising. Note that the y-axes differ across participants to appreciate the differences of the three methods for each individual participant.

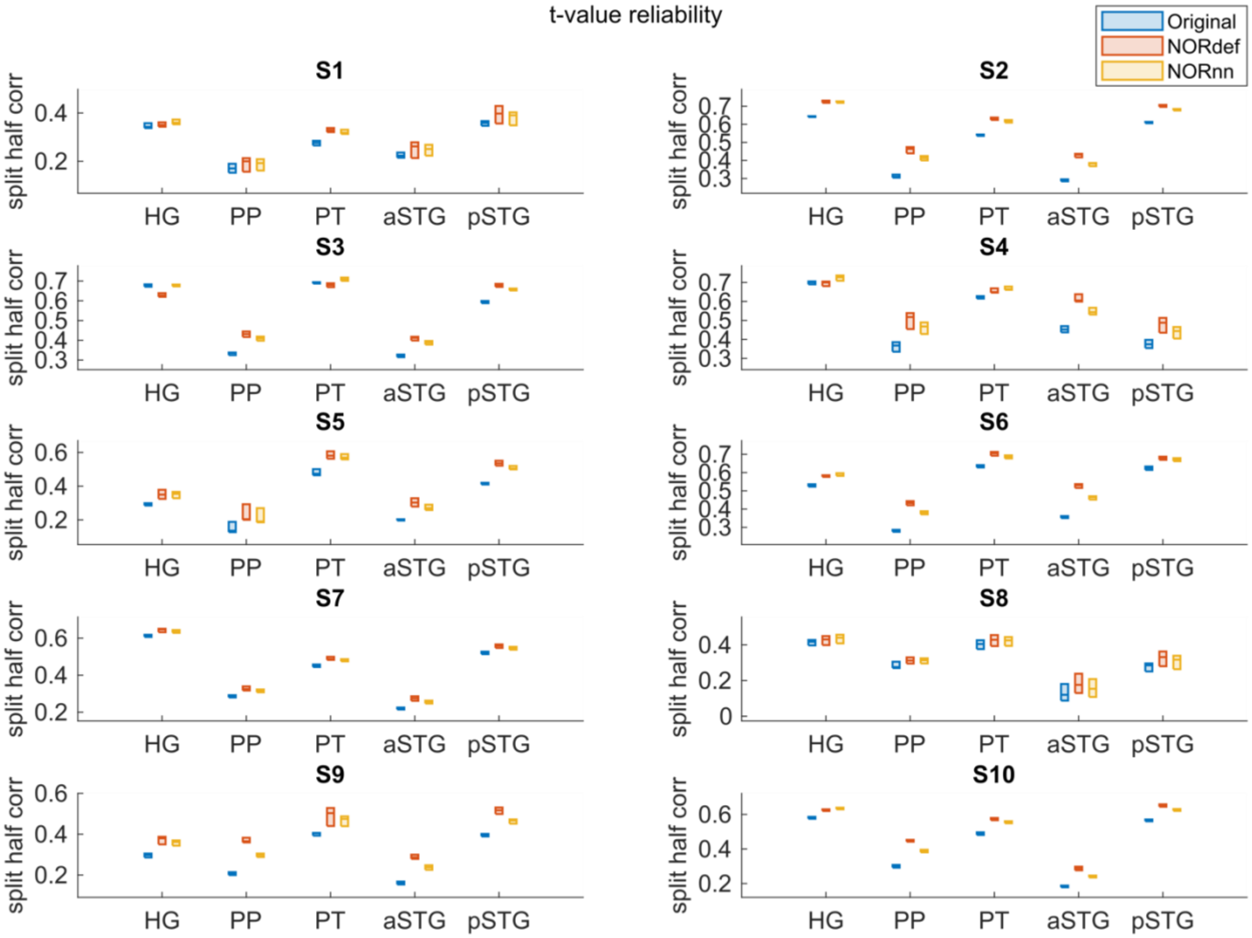

Figure S8. T-value reliability. This figure illustrates the data from all individual participants, as displayed in the group analysis of Figure 5D. T-values are generally more reliably estimated in NORDIC. Note that the y-axes differ across participants to appreciate the differences of the three methods for each individual participant.

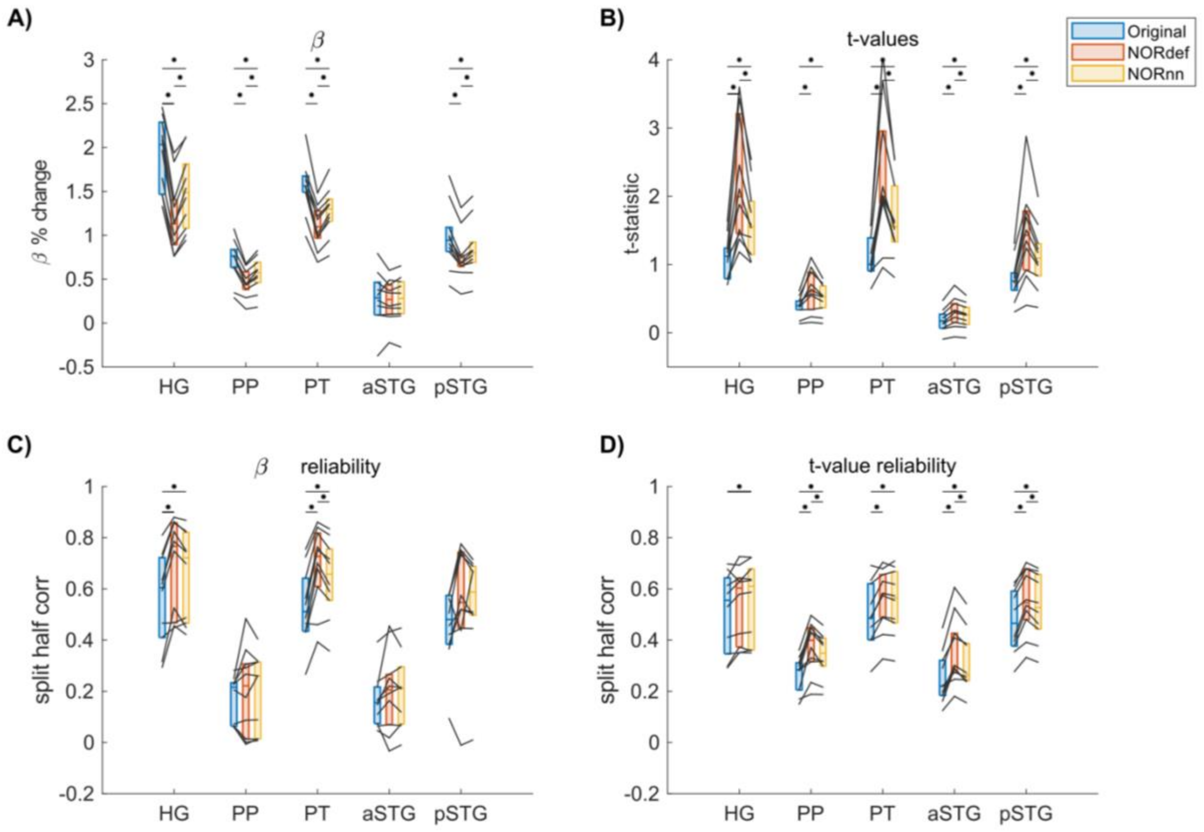

Figure S9. Beta- and t-value estimates and their reliability with individual subject lines. Same as Figure 5, but black lines indicate mean values of each individual subject. A) Average reduction of beta values across participants. B) At the group level, in increase in t-values is visible. C) On average, denoising results in a better estimate of beta values calculated with split half correlations in ROIs where there is more signal in the data. D) t-value reliability is generally higher after NORDIC than in the Original data. * indicates p<0.05.

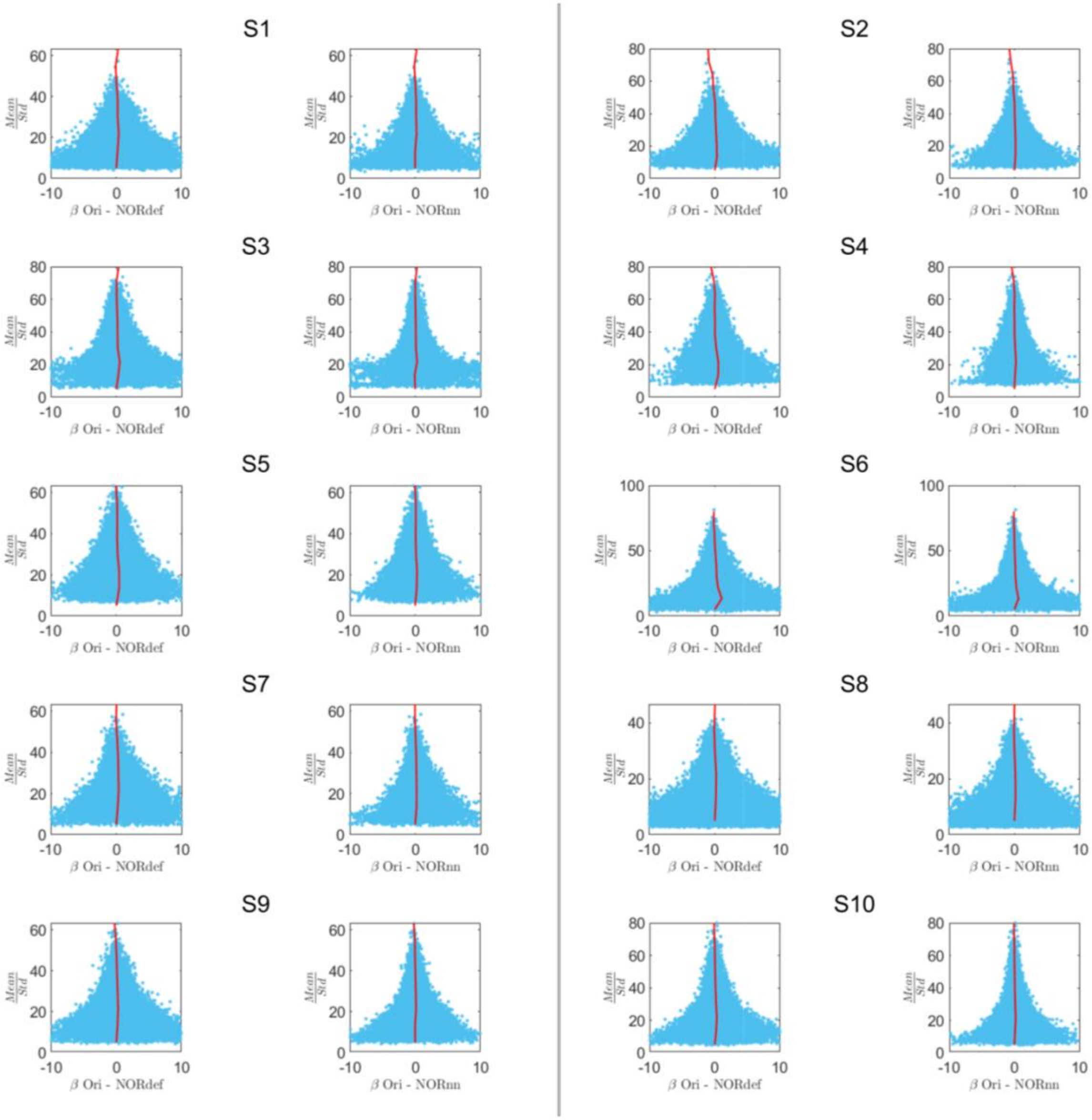

Figure S10. Beta difference in relation to tSNR for all subjects. Mean/standard deviation ($tSNR_{pr}$) is displayed as a function of the difference between the betas of the Original data and the betas after NORdef (left) and between the betas of the Original and NORnn data (right). For low $tSNR_{pr}$ values, the betas change in both directions. However, at high $tSNR_{pr}$, the betas remain relatively similar after NORDIC. The red line indicates the mean beta difference per bin. In all participants, we can see the same effect.

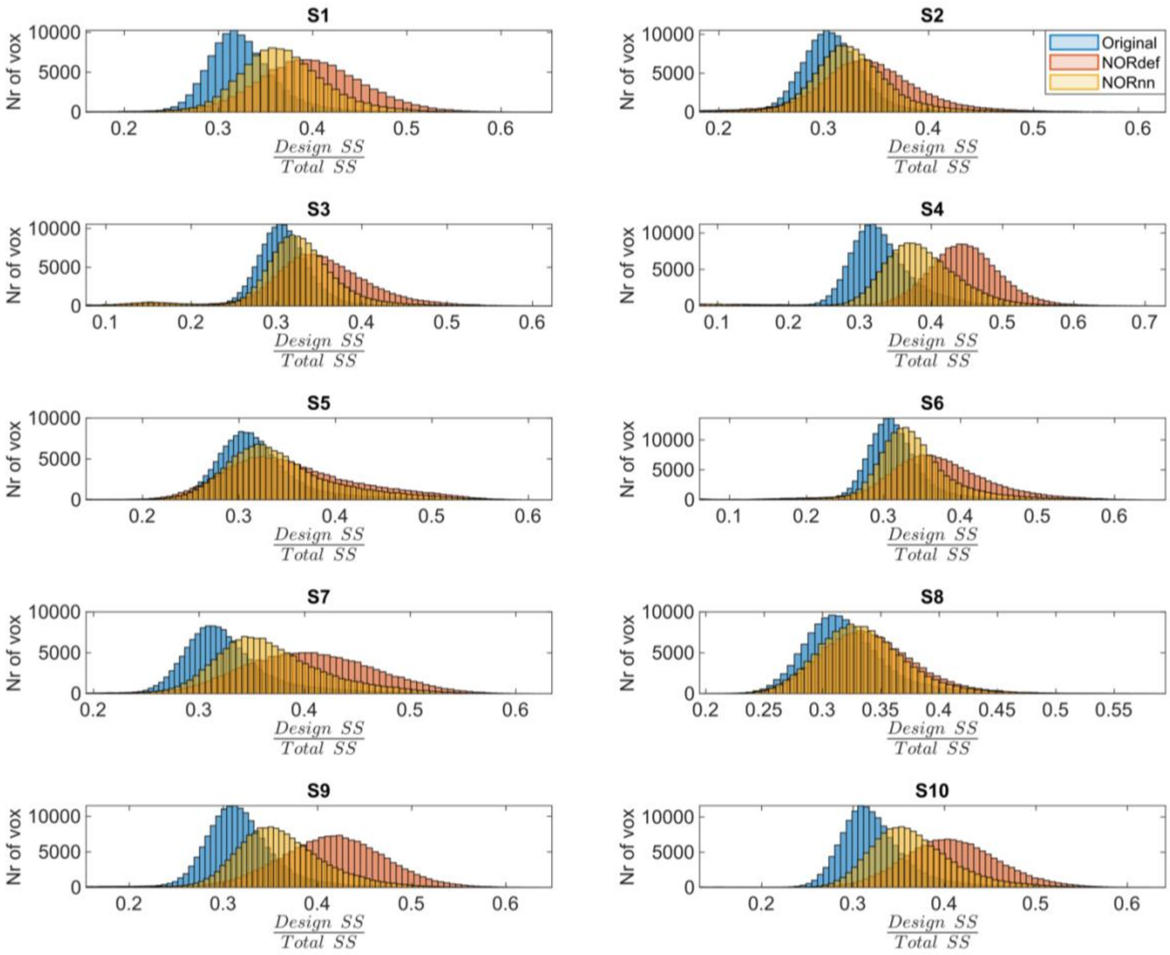

Figure S11. Proportion of variance explained by the design. This figure displays the individual plots (of which the group results are shown in Figure 7A). In general, the amount of variance explained by our design in the data increases consecutively with the use of NORnn and NORdef.

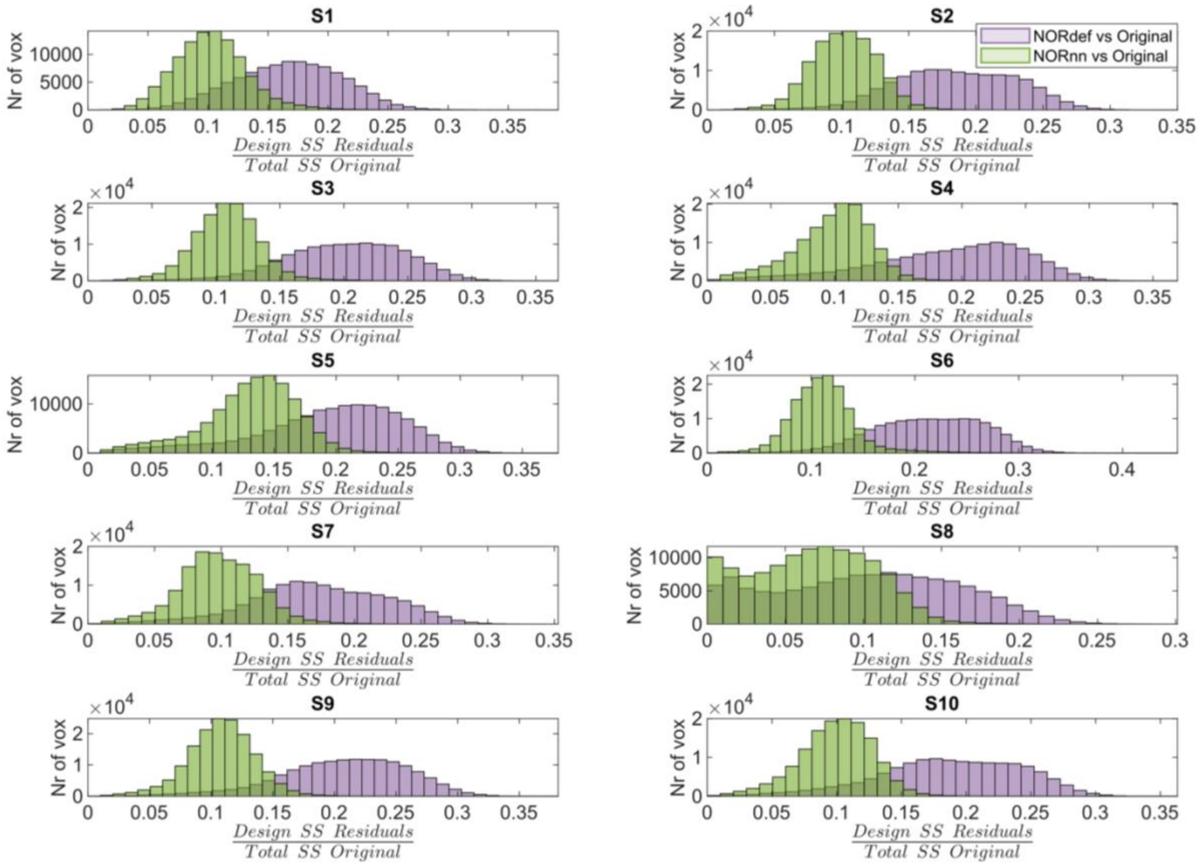

Figure S12. Removed proportion of variance explained by the design. This figure displays the individual plots (of which the group results are shown in Figure 7B). In general, denoising results in a partial removal of the signal of interest. A proportion of the variance in the residuals after NORDIC (see methods) can be explained by our stimulation design.

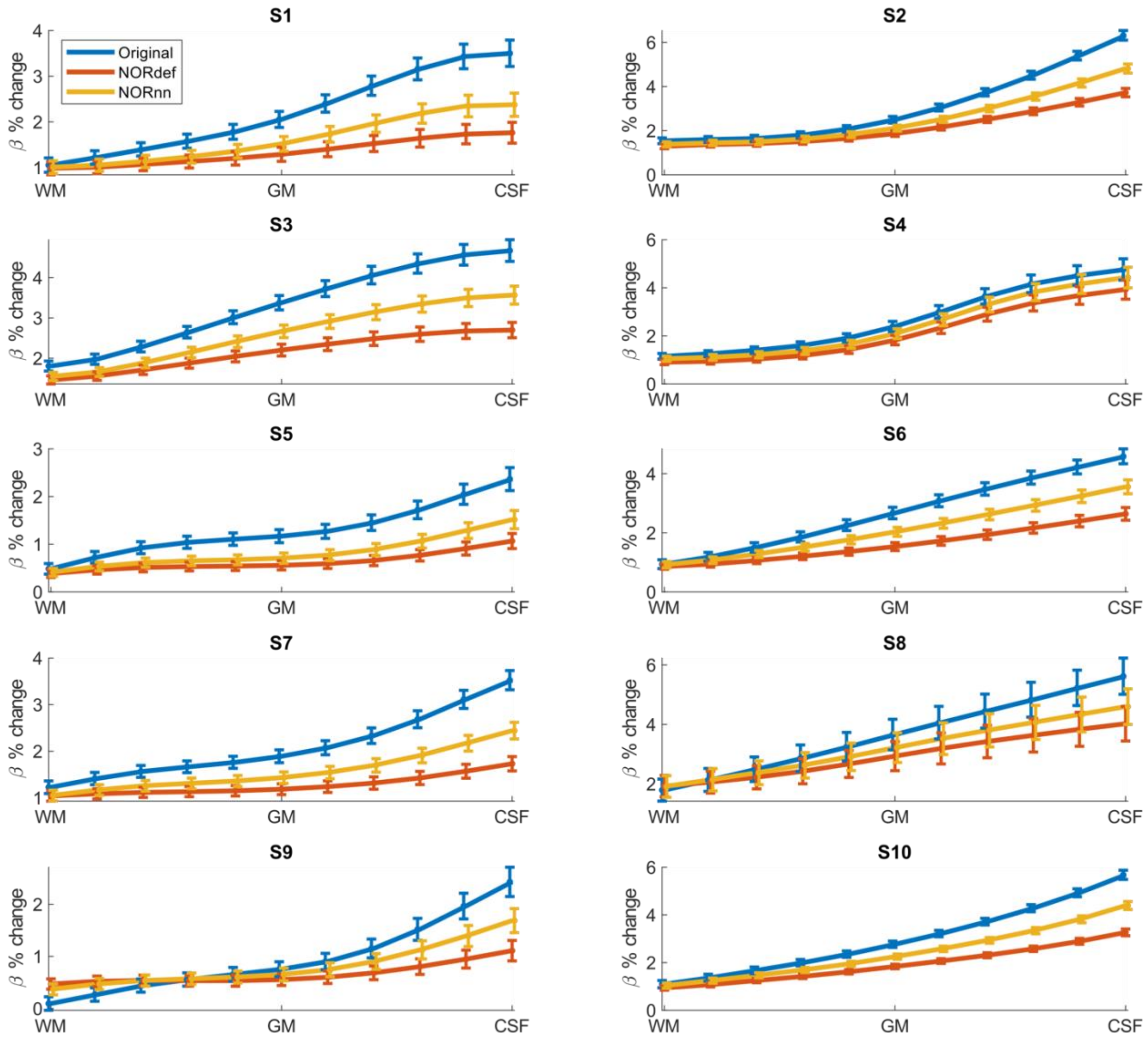

Figure S13. Effect of NORDIC across depth PredH. For all participants we plot the laminar response profiles for the PredH condition. In all plots we can easily identify the draining vein effect. However, we see a gradual decrease in slope for NORnn and NORdef, indicating that NORDIC denoising has a differential effect across depths. This trend is visible for all participants. Note that the y-axes differ across participants to appreciate the differences of the three methods for each individual participant.

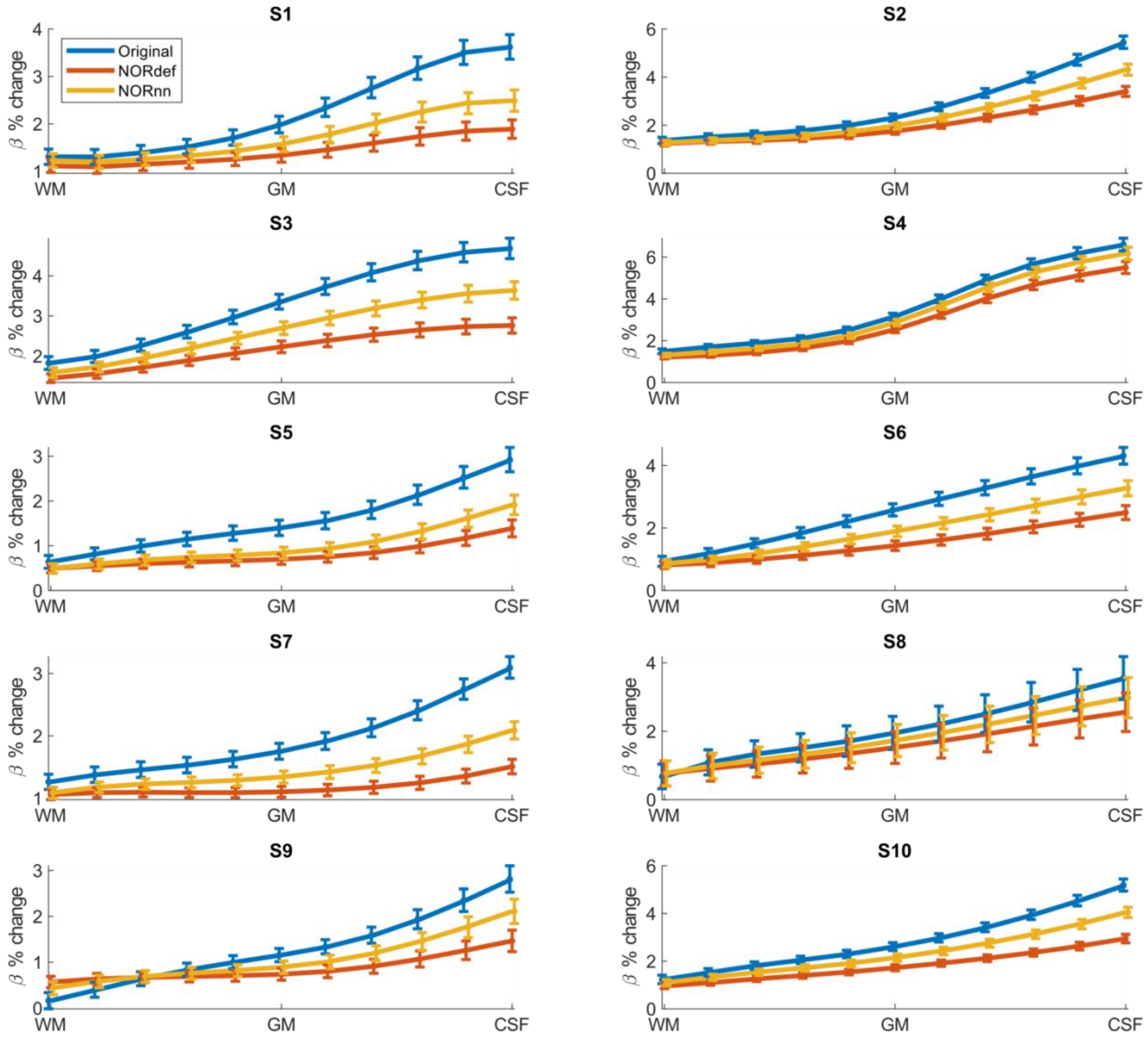

Figure S14. Effect of NORDIC across depth PredL. For all participants we plot the laminar response profiles for the PredL condition. Same as above, the draining vein effect can be observed in our GE-EPI Data. Similar to Figure S11, a gradual decrease in slope for NORnn and NORdef is visible, indicating that NORDIC denoising has a differential effect across depths. This trend is visible for all participants. Note that the y-axes differ across participants to appreciate the differences of the three methods for each individual participant.

Table 1.
P-values originating from all comparisons depicted in Figure 5. Correction for multiple comparisons using Bonferroni resulted in the lowest possible p-value being 0.015. Similarly, due to the Bonferroni correction, the p-value of certain comparisons is artificially higher than 1 (set to 1.000 in the table).

	**Original - NORdef**	**NORdef - NORnn**	**Original - NORnn**
Beta HG	0.015	0.015	0.015
Beta PP	0.015	0.015	0.015
Beta PT	0.015	0.015	0.015
Beta aSTG	1.000	1.000	1.000
Beta pSTG	0.015	0.044	0.015
t-value HG	0.015	0.015	0.015
t-value PP	0.015	0.103	0.015
t-value PT	0.015	0.015	0.015
t-value aSTG	0.015	0.015	0.015
t-value pSTG	0.015	0.015	0.015
Beta stab. HG	0.015	0.249	0.015
Beta stab. PP	1.000	1.000	1.000
Beta stab. PT	0.044	0.044	0.015
Beta stab. aSTG	1.000	1.000	1.000
Beta stab. pSTG	0.718	1.000	0.132
t-value stab. HG	0.660	1.000	0.015
t-value stab. PP	0.015	0.044	0.015
t-value stab. PT	0.044	1.000	0.015
t-value stab. aSTG	0.015	0.015	0.015
t-value stab. pSTG	0.015	0.015	0.015
